# Maf1 loss regulates spinogenesis and attenuates cognitive impairment in Alzheimer’s disease

**DOI:** 10.1093/brain/awae015

**Published:** 2024-01-16

**Authors:** Yingying Han, Kui Chen, Hongxiang Yu, Can Cui, Hongxia Li, Yongbo Hu, Bei Zhang, Gang Li

**Affiliations:** Department of Neurology, Shanghai East Hospital, School of Medicine, Tongji University, Shanghai 200092, China; Department of Neurosurgery, Xinhua Hospital, Shanghai Jiaotong University, School of Medicine, Shanghai 200092, China; Department of Neurology, Shanghai East Hospital, School of Medicine, Tongji University, Shanghai 200092, China; Department of Neurology, Shanghai East Hospital, School of Medicine, Tongji University, Shanghai 200092, China; Department of Neurology, Shanghai East Hospital, School of Medicine, Tongji University, Shanghai 200092, China; Department of Neurology, the First Affiliated Hospital of Naval Medical University (Shanghai Changhai Hospital), the Second Military Medical University, Shanghai 200092, China; Department of Neurology, Shanghai East Hospital, School of Medicine, Tongji University, Shanghai 200092, China; Department of Neurology, Shanghai East Hospital, School of Medicine, Tongji University, Shanghai 200092, China

**Keywords:** Alzheimer’s disease, synaptic plasticity, calcium homeostasis, Maf1, MNDAR1

## Abstract

Alzheimer’s disease is neurodegenerative and characterized by progressive cognitive impairment. Synaptic dysfunction appears in the early stage of Alzheimer’s disease and is significantly correlated with cognitive impairment. However, the specific regulatory mechanism remains unclear.

Here, we found the transcription factor Maf1 to be upregulated in Alzheimer’s disease and determined that conditional knockout of Maf1 in a transgenic mouse model of Alzheimer’s disease restored learning and memory function; the downregulation of Maf1 reduced the intraneuronal calcium concentration and restored neuronal synaptic morphology. We also demonstrated that Maf1 regulated the expression of NMDAR1 by binding to the promoter region of *Grin1*, further regulating calcium homeostasis and synaptic remodelling in neurons.

Our results clarify the important role and mechanism of the Maf1-NMDAR1 signalling pathway in stabilizing synaptic structure, neuronal function and behaviour during Alzheimer’s disease pathogenesis. This therefore serves as a potential diagnostic and therapeutic target for the early stage of Alzheimer’s disease.

## Teaser

Han, Chen *et al.* show that the transcription factor Maf1 is upregulated in the hippocampus of patients with Alzheimer’s disease and in APP/PS1 mice. Maf1 regulates the expression of NMDAR1 by binding to the promoter of the *Grin1* gene and could represent a potential therapeutic target for Alzheimer’s disease.

## Introduction

Alzheimer’s disease (AD) is a progressive neurodegenerative disease, which is often accompanied by memory loss, intellectual loss, social and emotional dysfunction and other symptoms. Pathological features include senile plaques formed by the aggregation of amyloid-β (Aβ) in the brain, neurofibrillary tangles (NFTs) formed by the aggregation of hyperphosphorylated tau (p-tau), chronic inflammation, loss of synapses and neuronal death.^1^ Recent studies have shown that in many patients with AD but without senile plaques and NFTs in the brain, the morphology and function of synapses are already significantly degraded and some neurons have died. Compared with amyloid plaque formation or neuron loss, synaptic loss is the most apparent morphological factor related to cognitive impairment in early AD, and synaptic degeneration has become a biological marker for the early stage of the disease.^2^ A recent study found that transcriptional regulation coordinates and regulates synaptic plasticity.^3^ Furthermore, studies have demonstrated that many transcription factors in the brain change significantly after AD onset^4-7^; however, their roles in early AD are still unclear.

*Maf1* is a eukaryote-specific gene in humans, rats, mice and lower animals such as *Drosophila* and silkworm, and it encodes a highly conserved protein.^8^ Maf1, first discovered in *Saccharomyces cerevisiae*, can be a transcriptional regulator of RNA polymerase III and plays an essential role in tumour suppression, glucose metabolism, lipid metabolism and other events by activating the PTEN pathway.^9^ In recent years, Maf1 has been found to bind and block RNA polymerase III-regulated transcription initiation and elongation. Moreover, it has been shown to regulate the activities of RNA polymerases I and II.^10^ Notably, the activity of Maf1 in yeast can be regulated by rapamycin-induced nutrient deficiency, DNA damage and secretory defects.^11^ Moreover, human Maf1 activity is altered in the context of DNA damage signalling and rapamycin-induced nutrient restriction.^12^ Maf1 can regulate the function of mitochondria,^13^ glucose metabolism,^14^ autophagy response^15^ and lipid metabolism,^16,17^ thus playing a role in tumour suppression,^18,19^ reproductive ability,^20^ obesity,^21^ growth and development^16,22^ and life expectancy,^15,23^ etc. Maf1 is highly expressed in the CNS, especially in the hippocampus and cortex.^24^ The latest studies have indicated that Maf1 can negatively regulate the growth of dendrites in hippocampal neurons through the PI3K-AKT-mTOR signalling pathway as well as dendritic spine growth, affecting learning and memory in mice *in vivo*.^25^ Additionally, a previous study demonstrated the neuroprotective effect of Maf1 on survival after root ganglion cell injury and provided a potential treatment strategy for traumatic optic neuropathy.^26^ As a result, Maf1 has become a hot topic, and it is therefore now possible to further clarify the mechanism of synaptic remodelling by exploring the morphology and potential function of Maf1 in regulating neuronal synapses.

In this study, we found the transcription factor Maf1 increased dramatically in the hippocampal tissue of a 6-month-old APP/PS1 transgenic mouse model and this was also observed in the hippocampus of AD patients, according to the Gene Expression Omnibus database, series GSE5281. Furthermore, we found that Maf1 regulated the expression of NMDAR1 by binding to the promoter region of the *Grin1* gene, further regulating calcium homeostasis and synaptic remodelling in neurons. Thus, our study reveals a Maf1-NMDAR1 pathway that causes synaptic and calcium deficits and provides a novel and potential therapeutic target for early AD.

## Materials and methods

### Animals

APP/PS1 [APP Swedish mutation (APPswe)/PSEN1dE9] transgenic mice on a C57BL6/J background [strain B6.Cg-Tg (APPswe, PSEN1dE9) 85Dbo/Mmjax] were obtained from Jackson Laboratory (Stock No. 005864; MMRRC Strain #034832-JAX).^27-29^ Maf1-eCKO1 transgenic mice were constructed by Shanghai Model Organisms. The *Maf1* gene was modified by flox through homologous recombination of fertilized eggs, based on the principle of homologous recombination, using CRISPR/Cas9-mediated gene editing in mice with a C57BL/6J background. The mice purchased from the company were flox heterozygous (Maf1^flox/+^). The flox heterozygous mice were self-bred to obtain flox homozygous mice (Maf1^flox/flox^). Tissue-specific infection by local injection of AAV2/9-Cre virus, coupled with neuron-specific promoters that drive the *Cre* gene, enabled stronger tissue-specific and neuron-specific gene recombination.

Heterozygous APP/PS1 mice were mated with C57BL/6J mice to obtain wild-type and APP/PS1 mice or mated with Maf1^flox/flox^ mice to obtain Maf1^flox/+^ and APP/PS1/Maf1^flox/+^ mice. The APP/PS1/Maf1^flox/+^ progeny were backcrossed with Maf1^flox/flox^ mice again, to obtain Maf1^flox/+^, Maf1^flox/flox^, APP/PS1/Maf1^flox/+^ and APP/PS1/Maf1^flox/flox^ mice (Maf1-eCKO1-APP/PS1). AAV-syn-Cre or AAV-syn-GFP was injected into the hippocampal CA1 region of wild-type and Maf1-eCKO1-APP/PS1 mice by stereotactic microinjection at 5 months, and the mice were subjected to the Morris water maze experiment after 1 month.

Institute of Cancer Research (ICR) mice were purchased from Shanghai JieSiJie Laboratory. Presenilin-1 M146V knock-in (PSEN1-M146V KI) mice were gifted from Prof. Suya Sun of Shanghai Jiaotong University.

To minimize behavioural variation, all mice were male, apart from the pregnant ICR mice used to extract the primary neurons. The animals were housed in a pathogen-free environment (23 ± 2°C, 45 ± 5% humidity, 12-h light/dark cycle), with *ad libitum* access to food pellets and water. All procedures involving animals were approved and monitored by Tongji University. All experiments complied with the National Institutes of Health Guidelines for the Care and Use of Laboratory Animals (Ministry of Health No. 55, revised 1998).

### Plasmid and viral construct generation

Three shRNA target sequences and one scrambled shRNA sequence (shSCR) were designed according to the *Maf1* gene, and the primers were synthesized as follows: shRNA1:TTGGAGAACTCCAGCTTTGAGGCCATCAA; shRNA2:TCTGCTTAGCTGAGTGTGACATCTACAGC; shRNA3:CCTCAATGAGTCCTTCCGGCCAGACTATG; and shSCR:GCACTACCAGAGCTAACTCAGATAGTACT. The shSCR did not target any gene and was used as a negative control. Maf1 overexpression plasmids (Maf1-OE) with a flag-tag were designed, and the control plasmid was an empty vector (NC). The single-stranded primers were annealed into double-stranded oligo sequences and linked into the double-digestion linearized RNA interference vectors. The correct transformant was verified by sequencing, and the high-purity plasmid was extracted. The Maf1 shRNA plasmid was constructed using the pGFP-CMV-ShLenti vector and further packaged into lentivirus. The Maf1 interference adeno-associated virus (AAV) was constructed using a pAAV2/9-hSyn-EGFP-3xFLAG-WPRE vector. The Cre interference AAV was constructed using a pAAV2/9-hSyn-EGFP-WPRE vector. APP695swe (K595N/M596L) virus was expressed from pSLenti-CMV vectors. The AAV serotypes mentioned later are AAV2/9 (abbreviated as AAV). Both the lentiviruses and AAVs were commissioned from Obio Technology and stored at −80°C.

### Cell cultures and transfection

Primary hippocampal cultures were prepared from embryonic Day 18 (E18) mouse brains.^30^ Neurons dissected from hippocampal tissue were inoculated with poly-D-lysine and cultured in neural base medium containing B27 supplement and GlutaMAX™. For lentivirus-infected neurons, at 6 days *in vitro* (DIV), concentrated lentivirus was added to the medium and transfected cells. The transfection efficiency was verified by fluorescence and the protein level after cell lysis. *In vitro* transfection of the APPswe virus at 6 DIV was followed by calcium phosphate transfection of shRNA plasmid at 2-day intervals and continued for 10 days to verify the regulation of neuronal dendrite growth by Maf1. When two plasmids were transfected simultaneously, the GFP-carrying plasmid was mixed with the other plasmid at a ratio of 1:3.

### Real-time quantitative PCR

Total RNA from primary hippocampal cultures was extracted using TRIzol reagent (Invitrogen). RNA was then reverse transcribed into cDNA using a Hifair^®^ RT Kit (Yeasen). Real-time quantitative PCR (RT-qPCR) was performed on a 7500 Real-Time PCR system (Applied Biosystems) using a Hieff Unicon^®^ qPCR SYBR Green Master Mix (Yeasen). The primer sequences used in this study can be found in Supplementary Table 1.

### Western blotting

Mouse hippocampus was dissected, homogenized and solubilized at 4°C for 30 min in lysis buffer. Lysates were centrifuged at 12 000*g* for 20 min at 4°C to remove the insoluble deposits, and the protein concentrations were estimated using a bicinchoninic acid (BCA) protein assay kit (Thermo Fisher Scientific). Then, the protein was boiled in sodium dodecyl sulphate (SDS)-loading buffer at 95°C for 10 min for denaturation. Proteins were run on 10% polyacrylamide gels and transferred to nitrocellulose membranes. Membranes were blocked in Tris-buffered saline and Tween 20 (TBST; 150 mm NaCl, 10 mm Tris, 0.1% Tween 20, pH 7.6) containing 5% bovine serum albumin (BSA) for 1 h at room temperature. The primary antibodies were diluted in blocking buffer and incubated overnight at 4°C. After washing with TBST three times, the blots were incubated with horseradish peroxidase (HRP)-conjugated secondary antibodies for 1 h at room temperature. After another three washes, blots were exposed to enhanced chemiluminescence substrate. Quantifications were performed by analysing the relative densities of exposed film using ImageJ. For primary antibodies, we used rabbit anti-Maf1 (1:500, Abcam, Cat. No. ab230499), rabbit anti-β-actin (1:1000, Cell Signaling Technology, Cat. No. 8457S), mouse anti-GAPDH (1:3000, Invitrogen, Cat. No. MA5-15738-D680), rabbit anti-NMDAR1 (1:1000, Abcam, Cat. No. ab109182), mouse anti-NMDAR1 (1:1000, Abcam, Cat. No. ab134308), rabbit anti-amyloid-β 6E10 (1:1000, Novusbio, Cat. No. NBP2-62566), immunoprecipitation-rabbit IgG (1:1000, Abcam, Cat. No. ab172730), rabbit anti-NMDAR2A (1:1000, Abcam, Cat. No. ab227233) and mouse anti-NMDAR2B (1:1000, BD, Cat. No. 610417).

### Immunohistochemistry and immunofluorescence

Primary hippocampal neurons were fixed in PBS containing 4% paraformaldehyde (PFA) for 30 min and stored at 4°C. Mice were perfused and brains were fixed with 4% PFA solution at 4°C in PBS overnight. Coronal brain slices (30-μm thick) were generated using a VT1000 vibratome (Leica Biosystems). For immunohistochemistry (IHC), 3% H_2_O_2_ was used to block endogenous peroxidase activity for 10 min at room temperature and then blocking solution: 0.03% Triton X-100 (MilliporeSigma) and 10% donkey serum (Invitrogen) in PBS was applied for 10 min at room temperature. The sections were then incubated with primary antibody overnight at 4°C in antibody solution: 0.03% Triton X-100 and 2% donkey serum in PBS. Next, the sections were incubated with enzymic anti-rabbit antibodies and stained with a DAB kit (Vector Labs). For immunofluorescence (IF), sections were blocked with blocking solution for 30 min and incubated with primary antibody and cell nuclei stained with DAPI overnight in antibody solution. Slices were rinsed in PBS for 10 min three times and then incubated with Alexa Fluor secondary antibodies for 2 h at room temperature. Slices were rinsed in PBS for 10 min three times and the chamber slides were then mounted with mounting medium and imaged. The following antibodies were used: rabbit anti-Maf1 (1:150, Abcam, Cat. No. ab230499), chicken anti-Map2 (1:10 000, Abcam, Cat. No. ab5392) and Alexa Fluor secondary antibodies (Alexa Fluor 488, Alexa Fluor 555, Alexa Fluor 647; 1:500; Thermo Fisher Scientific).

### Image analysis and quantification

Confocal microscopy (Leica SP8) was used to obtain high-resolution images of dual-immunofluorescence experiments. A *z*-series of 7–12 images with a 0.5–1 μm depth interval, each averaged two times, was taken at a resolution of 1024 × 1024 pixels. The resultant stack was flattened into a single image using a maximum intensity projection.^31^ A 20× objective was used to measure the fluorescence intensity of the target protein and cell localization. For the analysis of dendritic spines, neurons were imaged with a 63× objective and 2× zoom with 1024 × 1024 pixel resolution. GFP-positive cells were used to quantify and classify neuronal spines.^32^ The cells were quantified using ImageJ.

### RNA-sequencing and data analysis

Total RNA was extracted using a mirVana miRNA Isolation Kit (Ambion) following the manufacturer’s protocol. RNA integrity was evaluated using an Agilent 2100 Bioanalyzer (Agilent Technologies). The samples with an RNA integrity number (RIN) ≥ 7 were used for subsequent analyses. Libraries were constructed using a TruSeq Stranded mRNA LTSample Prep Kit (Illumina) according to the manufacturer’s instructions and sequenced on an Illumina sequencing platform (HiSeqTM 2500 or Illumina HiSeq X Ten), generating 125 bp/150 bp paired-end reads. The transcriptome sequencing and analysis were conducted by OE Biotech Co. Ltd. (Shanghai, China).

### Cleavage Under Targets and Tagmentation analysis

Hippocampal tissue was separated from the brain after anaesthesia, and lysate was added to promote the rapid release of the nuclei, before Concanavalin A magnetic beads resuspended in binding buffer were added. The target protein was bound with the primary antibody and incubated with the secondary antibody. Next, ChiTag transposition was used to bind antibodies, and ChiTag transposition was activated to fragment the target DNA. Finally, DNA was extracted, PCR was performed and DNA was purified and sequenced. Shanghai Ouyi Biomedical Technology Co. Ltd. assisted with the sequencing and data analysis.

### Co-immunoprecipitation assays

Primary hippocampal neurons were lysed to obtain protein extracts using Thermo Scientific^™^ Pierce^™^ IP Lysis Buffer (Thermo Fisher Scientific, Cat. No. 87787) containing PMSF. One microgram of Maf1 antibody or NMDAR1 antibody was added to protein extracts and left to react overnight at 4°C following incubation with protein G beads for 3 h at 4°C. Immunoblot experiments were conducted and tested with the indicated antibodies.

### Chromatin immunoprecipitation assay

A chromatin immunoprecipitation (ChIP) assay was conducted using a Pierce^™^ Agarose ChIP Kit (Thermo Fisher Scientific) according to the manufacturer’s instructions. Chromatin was extracted, and DNA was cut to 0.2-kb to 1-kb fragments. Primary hippocampal neurons were cross-linked using 1% formaldehyde, and the chromatin was immunoprecipitated with the antibody Maf1 (Abcam). IgG was used as a negative control. DNA was re-suspended in 50 μl of Tris-EDTA buffer and amplified by PCR. PCR products were determined on a 1.5% agarose gel. DNA was purified (TransGen Biotech, Cat. No. EP101) and further analysed with qPCR using the primers against the *Grin1* promoter. The ChIP-PCR primer sequences used in this study can be found in Supplementary Table 1.

### Dual-luciferase reporter assay

A dual-luciferase reporter assay was used to detect the interaction between the Maf1 and *Grin1* promoter regions. The pGL3 promoter vector was used as the plasmid. The Maf1 overexpression plasmid and the wild-type and mutant *Grin1* promoter luciferase reporter constructs were generated by Genomeditech Co. Ltd. Cells were co-transfected with the wild-type or mutant (MT) plasmids containing Maf1 or a negative control using Lipofectamine 2000 (Invitrogen). Forty-eight hours post-transfection, a Dual-Luciferase^®^ Reporter Assay System (Promega) was used to measure the relative luciferase activity—firefly luciferase activity was normalized to Renilla luciferase activity. Experiments were performed in triplicate. The mutation constructs on the *Grin1* promoter were at −1774 to 951 (MT), −1774 to −1697 (MT1), −1308 to −1217 (MT2) and −1039 to −951 (MT3).

### Calcium imaging

Primary hippocampal neurons were treated with APPswe lentivirus and shMaf1 lentivirus at 8 DIV. Calcium imaging experiments were conducted at approximately 14–16 DIV. Neurons were incubated for 30 min at 37°C with 2 μmol/ml Fura-4 AM (Beyotime Biotechnology, Cat. No. S1061M). After being washed three times with physiological saline, cells were excited at 340 nm and observed at 510 nm with an inverted fluorescence microscope.

### Amyloid-β ELISA

The contents of Aβ_40_, Aβ_42_ and β-CTF in mouse hippocampus (*n* = 8 for each age group) were measured using ELISA kits (Jiangsu Jingmei Biological Technology, Cat. Nos. JM-11861, 11863, 13038) according to the manufacturer’s instructions.

### Stereotaxic injection

Mice were anaesthetized using 1% sodium pentobarbital solution (50 mg/kg, intraperitoneal). Holes were drilled above the CA1 field of the hippocampus (anterior/posterior = ±1.25 mm, medial/lateral = ±1.7 mm, dorsal/ventral = ±1.6 mm). AAV2/9-syn-shSCR-GFP, AAV2/9-syn-shMaf1-GFP, AAV-syn-GFP vector or AAV-syn-Cre-GFP (10^12^ IU/ml, 2 μl) were bilaterally microinfused into the hippocampus via a cannula connected to a Hamilton microsyringe. The infusion rate was 0.2 μl/min, and the cannula was left in place for 10 min following completion of the infusion.

### Morris water maze

Based on a previous study,^33^ the Morris water maze (MWM) test was conducted. Briefly, learning and memory function were assessed 1 month after AAV2/9 injection. Recording was started during the acquisition phase. If the mice found the platform and remained there for ≥3 s, recording stopped automatically. For mice that did not find the platform within 60 s, the incubation period was denoted as 60 s, and the mice were guided to stay on the platform for 20 s. In the space exploration stage of Day 6, the circular platform in the target quadrant was removed. The mice were allowed to swim in the water for 60 s and parameters such as the number of times the mice crossed the platform, the total distance moved and the percentage of time spent in each quadrant were recorded. The ANY-maze automated video system (MED Associates) was used to record all the activity of mice through a camera video, and the results were collected and calculated for statistical analysis to measure the latency in reaching the platform, the percentage of time spent in the target quadrant and the number of times the mice traversed the platform.

### Golgi staining

Mice were anaesthetized using 1% sodium pentobarbital solution (50 mg/kg, intraperitoneal). After immersion in mordant solution for 3 days, followed by immersion in 1.5% AgNO_3_ solution at room temperature for 5 days in a dark environment, the brain tissue was dehydrated in a graded ethanol series (70%, 2 h; 80%, 2 h; 90%, overnight; 95%, 90 min × 2; and 100%, 40 min × 2), cleared in xylene (20 min × 2), embedded in paraffin and cut serially into 60 µm sections. Using digital images at ×1000 magnification, 15 or more fully impregnated neurons from each group of mice were randomly selected from areas not obscured by adjacent neurons. The dendritic spine density (number/µm) was calculated using ImageJ.

### Electrophysiological recording of brain slices

Brain was rapidly disassembled and frozen in cryogenic artificial CSF (ACSF) containing (in mM):125 NaCl, 2.5 KCl, 2 CaCl_2_, 1 MgCl_2_, 25 NaHCO_3_, 1.25 NaH_2_PO_4_ and 12.5 glucose. Coronal brain sections (300-μm thick) were prepared using a vibrator and soaked at 31°C for 1 h in ACSF containing 95% O_2_ and 5% CO_2_ and then stored at room temperature (22°C–25°C). The micro excitatory postsynaptic potential (mEPSP) at −70 mV was recorded in the presence of 100 μM microtoxin and 1 μM tetrodotoxin. To record long-term potentiation (LTP), extracellular field EPSPs (fEPSPs) in the Schaffer collateral pathway were evoked synaptically and recorded in the CA1 region. LTPs are induced by high-frequency stimuli consisting of 1-s 100 Hz sequences, each with an intensity of 70%–80% that causes peak fEPSPs. Data were analysed in pCLAMP 10.6 (Molecular Devices) with an average of three cells per sheet and two to three sheets recorded per mouse.

### Statistical analysis

All statistical analyses were performed in GraphPad Prism 6.01. Data are presented as mean ± standard error of the mean from at least three biological replicates for experiments. One-way or two-way ANOVA followed by Dunnett’s test, or *t*-tests were used. Non-normally distributed data were analysed using the Kruskal–Wallis test. Differences between groups were judged to be statistically significant when *P* < 0.05.

### Ethics statement

The animal study was reviewed and approved by Shanghai East Hospital, School of Medicine, Tongji University.

## Results

### Maf1 expression is elevated in the hippocampus of Alzheimer’s disease patients and APP/PS1 mice

**Figure 1:**
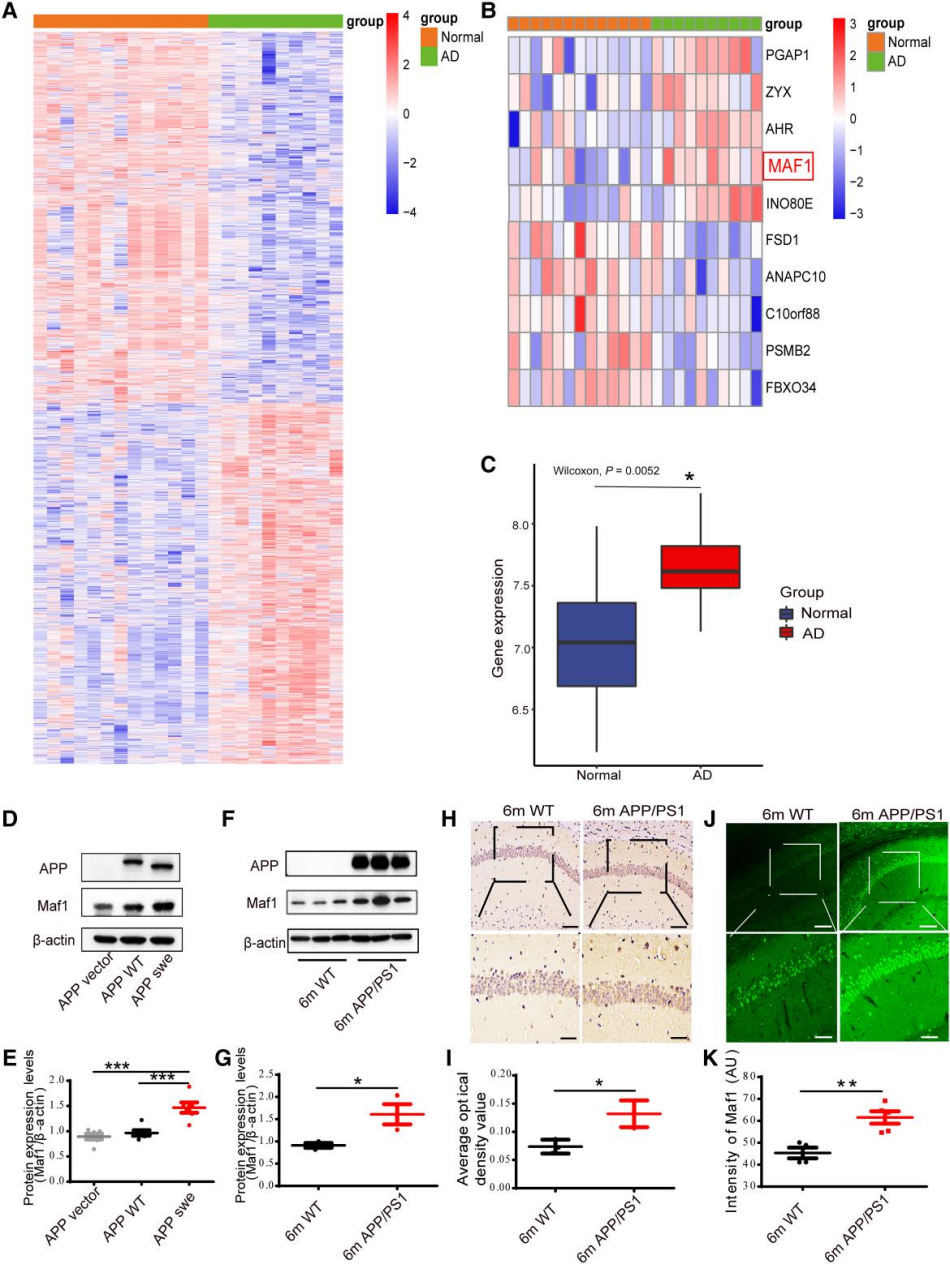
**Increased expression of Maf1 in Alzheimer’s disease patients and APP/PS1 mice**. (**A**) Heat map of differentially expressed genes in Alzheimer’s disease (AD) and normal human brain from GEO database GSE5281. (**B**) Heat maps of Maf1 expression in normal subjects and AD patients from the GEO database. (**C**) The expression of Maf1 in human brain was elevated in AD patients compared to corresponding non-demented controls (*n* = 13 and *n* = 10, respectively). (**D** and **E**) Quantitative western blot analysis of primary hippocampal neurons, revealing an increase in Maf1 in mouse hippocampus transfected with APPswe virus compared with APP vector and APP wild-type (WT). (**F** and **G**) Levels of Maf1 protein in hippocampal neurons of APP/PS1 mice aged 6 months compared with wild-type mice. *n* = 4 mice per group. (**H** and **I**) Immunohistochemical results for Maf1 in APP/PS1 mice aged 6 months. *n* = 4 mice per group. (**J** and **K**) The results of Maf1 immunofluorescence in APP/PS1 mice aged 6 months. Data are presented as mean ± standard error of the mean. *P < 0.05; **P < 0.01; ***P < 0.001.

Memory decline and loss of cognitive ability in AD are directly related to the morphology and function of neurons. Based on previous studies, we considered that Maf1, as a transcription factor, might regulate the existence of other genes in the pathogenesis of AD. By referring to the GEO database, we found that Maf1 expression was significantly raised in AD (*P* = 0.007256926; Fig. 1A–C).

We further verified whether this phenomenon existed in AD models *in vitro* and AD transgenic mice. To explore the expression of Maf1 in hippocampal neurons, primary hippocampal neurons extracted from the hippocampus of fetal mice were used as an *in vitro* AD model. The purity of the extracted neurons was >90% according to NeuN staining (Supplementary Fig. 1A). We also explored the expression of Maf1 in hippocampal neurons *in vivo* and *in vitro* using immunofluorescence and found that Maf1 and MAP2 (neuronal marker) were co-labelled in both brain slices and *in vitro* primary neurons, indicating that Maf1 was expressed in both the cell body and dendrites of hippocampal neurons (Supplementary Fig. 1B), consistent with previous studies. Following an *in vitro* assay, we noted that Maf1 protein levels were significantly elevated in primary hippocampal neurons transfected with APPswe virus compared with primary neurons transfected with control virus (Fig. 1D and E). Furthermore, we found that the level of Maf1 protein was also significantly increased in hippocampus of 6-month-old APP/PS1 mice (Fig. 1F and G). However, Maf1 was not elevated in the hippocampus of PSEN1-M146V KI mice (Supplementary Fig. 1C and D). We confirmed the increased expression of Maf1 protein in hippocampus of APP/PS1 mice by immunohistochemistry and immunofluorescence (Fig. 1H–K). Moreover, we found that the karyotoplasmic ratio of Maf1 in AD was significantly increased through statistical localization of Maf1 in the nucleus and cytoplasm (Supplementary Fig. 1E). Thus, these data indicated that Maf1 expression levels are abnormally elevated in AD.

### Maf1 conditional knockout in hippocampal neurons improves learning and memory function

Given previous studies indicating that Maf1 can impair learning and memory abilities in mice, we asked whether decreased expression of Maf1 could restore these abilities in AD mice. First, we used the CRISPR/Cas9 system to generate Maf1 conditional knockout mice (Maf1-eCKO1) to investigate the impact of deletion in the hippocampal neurons of AD mice. The guide RNA was designed to target a specific exon to create Maf1-eCKO1 mice, and the transgenic mice were crossbred with APP/PS1 mice to obtain Maf1-eCKO1-APP/PS1 mice (Supplementary Fig. 2A and B). Genotyping was performed from mouse tail tips, and the homozygous *Maf1* gene flox and APP/PS1 mice that could be subjected to conditional knockout were confirmed by PCR (Supplementary Fig. 2C). We injected AAV-syn-Cre or AAV-syn-GFP into the hippocampal CA1 region of wild-type and Maf1-eCKO1-APP/PS1 mice with stereotactic microinjections at 5 months (Supplementary Fig. 2D and E). One month later, the mice underwent a 5-day MWM learning and acquisition experiment, and a probe test was performed on Day 6 to measure the mice’s ability to locate quadrants that previously contained hidden platforms (Supplementary Fig. 2E). After injection of AAV-hsyn-Cre-GFP into the hippocampus, the deletion of Maf1 protein was confirmed by western blot analysis and immunofluorescence (Supplementary Fig. 2F and G). Compared with Maf1-eCKO1-APP/PS1 mice injected with control virus, Maf1 bands were significantly reduced in Maf1-eCKO1-APP/PS1 mice injected with AAV-syn-Cre-GFP (Supplementary Fig. 2F).

**Figure 2:**
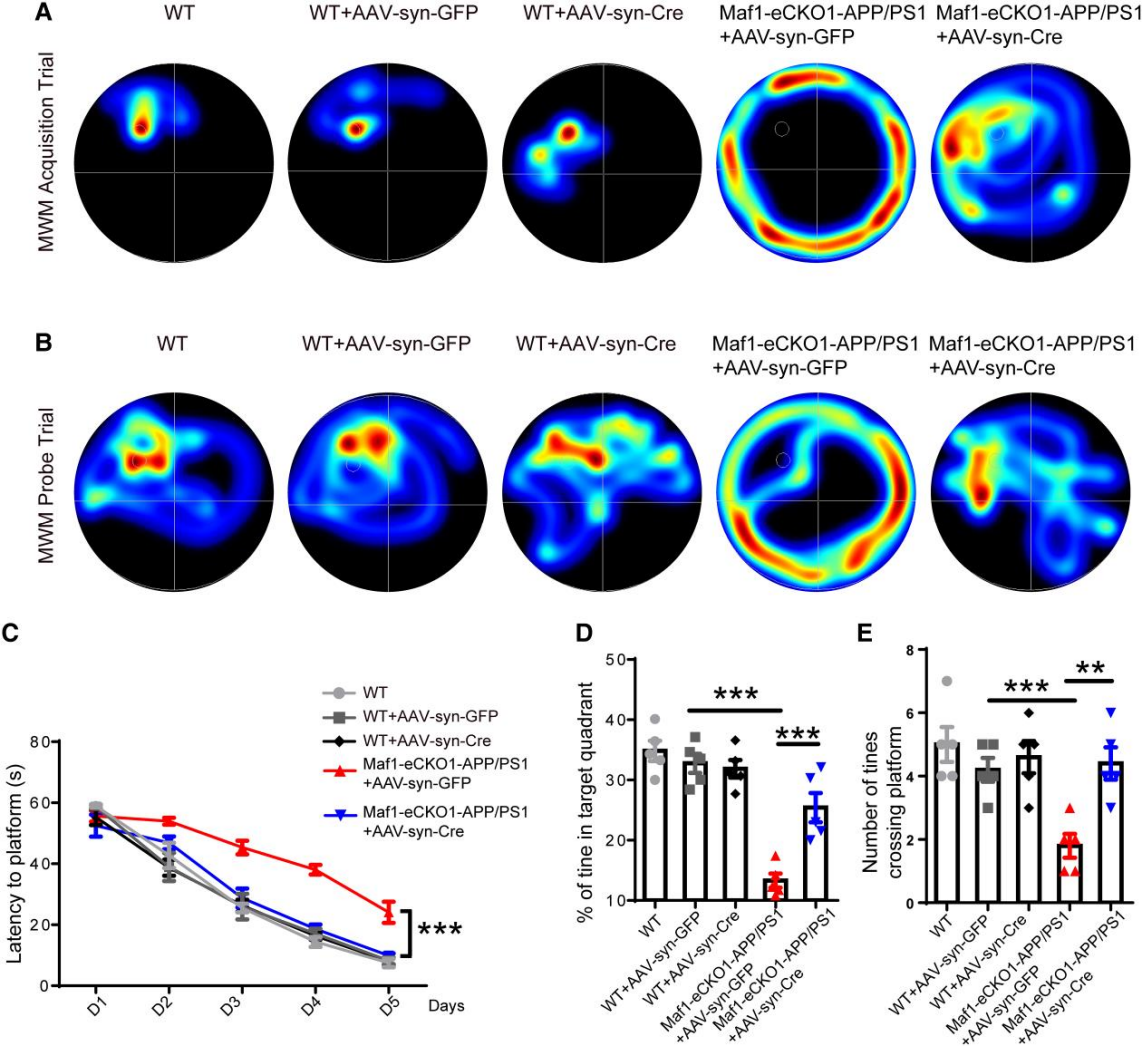
**Maf1 conditional knockout can improve learning and memory function in Alzheimer’s disease mice**. (**A**) Representative plots of the Morris water maze (MWM) trajectories of each group of mice on Day 5 of the training acquisition stage. (**B**) Representative plots of each group of mice on Day 6 of the space exploration phase of the MWM test. (**C**) Latency in finding the escape platform was measured on Days 1–5 of the training acquisition phase. (**D** and **E**) The percentage of time spent in the target quadrant and the number of times the target platform area was crossed during the space exploration phase. *n* = 5 mice per group. Data are presented as the mean ± standard error of the mean. *P < 0.05, **P < 0.01,***P < 0.001.

After verifying the effectiveness of the conditional knockout, we performed a MWM behaviour test to determine the effect of Maf1 expression in the hippocampus on learning and memory. During the acquisition test, there was no difference between the three groups of wild-type mice treated differently, while the average escape latencies on training Days 2, 3, 4 and 5 in the Maf1-eCKO1-APP/PS1+AAV-syn-Cre mice were significantly shorter compared with the Maf1-eCKO1-APP/PS1+AAV-syn-GFP mice (Fig. 2A and C). Moreover, the conditional knockout of Maf1 resulted in reduced latency on Day 5 in Maf1-eCKO1-APP/PS1+AAV-syn-Cre mice compared with Maf1-eCKO1-APP/PS1+AAV-syn-GFP mice (Fig. 2A and C).

According to the probe test, there was no difference in the percentage time spent in the target quadrant between the three groups of wild-type mice treated differently. Wild-type and Maf1-eCKO1-APP/PS1+AAV-syn-Cre mice swam mainly in or near the target quadrant. In contrast, Maf1-eCKO1-APP/PS1+AAV-syn-GFP mice swam less near the target quadrant (Fig. 2B). Compared with the Maf1-eCKO1-APP/PS1+AAV-syn-GFP mice, the Maf1-eCKO1-APP/PS1+AAV-syn-Cre- mice spent more time in the target quadrant (Fig. 2D). Furthermore, the number of platform crossings performed by the Maf1-eCKO1-APP/PS1+AAV-syn-Cre mice was significantly higher than by the Maf1-eCKO1-APP/PS1+AAV-syn-GFP mice (Fig. 2E). Taken together, these results indicated that Maf1 is important for cognitive function in AD.

### Maf1 contributes to neural synaptic defects and calcium signals

**Figure 3:**
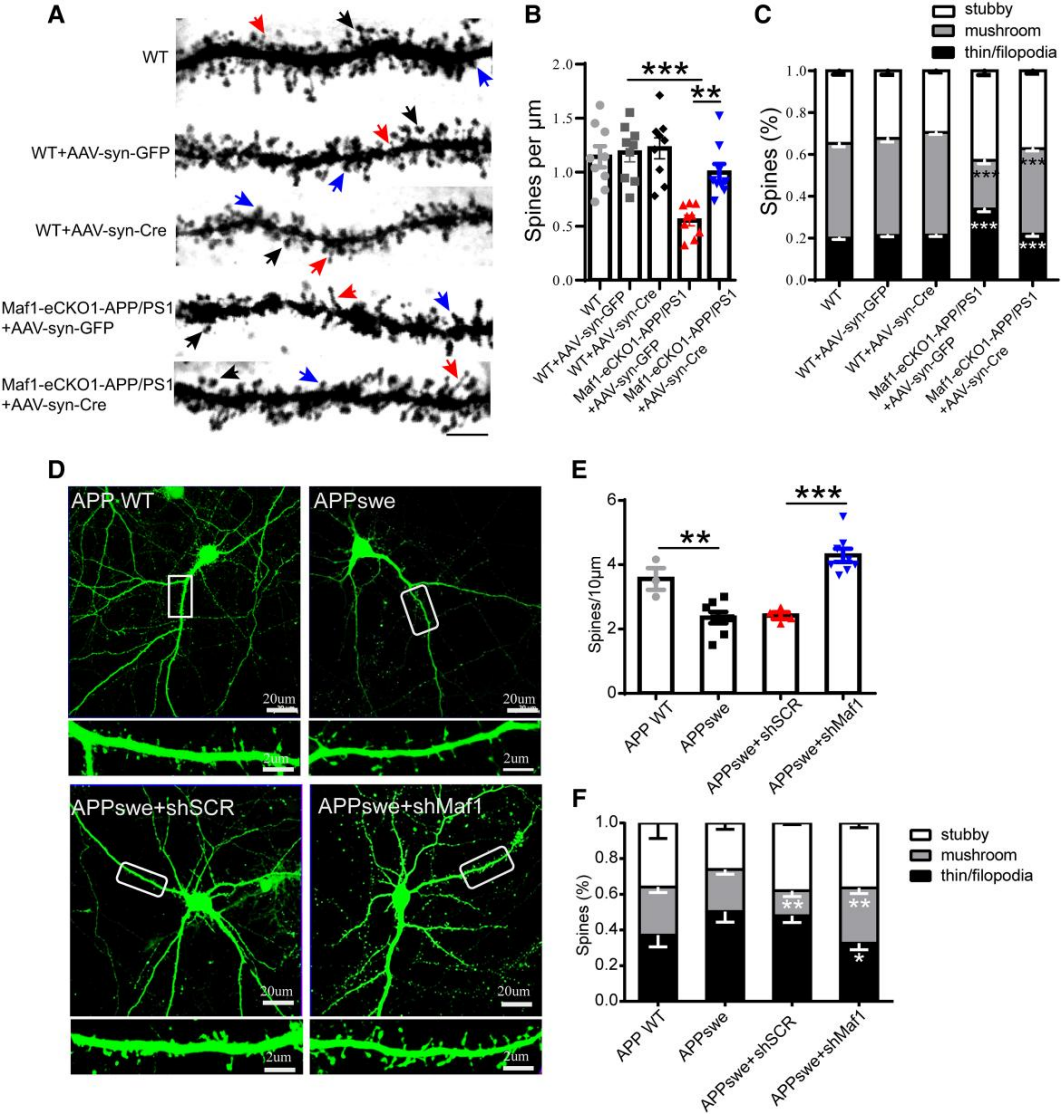
**Maf1 conditional knockout in hippocampal neurons promotes the maturation of neuronal dendritic spines in Alzheimer’s disease**. (**A**) Golgi staining of mouse hippocampal neurons. The morphology and quantity of dendritic spines were analysed under ×100 magnification. The black arrows indicate mushroom-like spines, red arrows indicate thin/filopodia spines and blue arrows indicate stubby spines. Scale bar = 10 μm. (**B**) Statistical analysis of the density of neuronal dendritic spines in the groups of mice described in **A**. *n* = 9 neurons for each group. (**C**) Statistical diagram of the classified percentage of neuronal dendritic spines in the groups of mice described in **A**. *n* = 9 neurons for each group. (**D**) GFP fluorescence after transfection of primary hippocampal neurons with shSCR and shMaf1 plasmids. The areas surrounded by white boxes are enlarged below each image. Scale bars = 20 μm (*top*); 2 μm (*bottom*). (**E**) Statistical analysis of the density of neuronal dendritic spines *in vitro*. (**F**) Statistical diagram of classified percentages of neuronal dendritic spines *in vitro*. *n* = 3–6 neurons for each group. Data are presented as mean ± standard error of the mean. *P < 0.05, **P < 0.01, ***P < 0.001.

Synaptic loss and dysfunction lead to the decline of cognitive function in patients with AD. This is manifested by the reduction in the number of mushroom dendritic spines and the decline of learning and memory etc., and these changes in the morphology and essential functions of nerve cells lead to the loss of cognitive function in AD patients.^34^ Notably, synapse structure, number and function are crucial to maintaining brain function. Therefore, we examined spine density using Golgi-Cox staining (Fig. 3A) to test whether the knockdown of Maf1-induced behavioural recovery was associated with structural changes in dendritic spine density *in vivo*. The Maf1-eCKO1-APP/PS1+AAV-syn-Cre mice showed an 18% increase in the density of spines compared with the Maf1-eCKO1-APP/PS1+AAV-syn-GFP mice (Fig. 3B). The density of mushroom-like spines also increased by about 10%, and the density of thin/filopodia-like spines decreased by about 17% (Fig. 3C). These results suggest that a decrease in Maf1 can significantly restore dendritic spine injury of hippocampal neurons in AD.

To verify this phenomenon *in vitro*, we first verified the effectiveness of the constructed plasmid and virus. Following the transfection of lentivirus into HEK293T cells, a higher GFP fluorescence intensity was observed after 72 h (Supplementary Fig. 3A). ShRNAs targeting Maf1 effectively reduced the endogenous protein levels compared with shSCR-transfected neurons, with shMaf1-2 having the best effect (Supplementary Fig. 3B). In addition, a significant increase in the expression of Maf1 and the presence of labelled protein were detected by western blot, which confirmed the successful overexpression of Maf1 (Supplementary Fig. 3C). ShSCR, shMaf1 and Maf1-OE lentiviruses were further used to transfect mature primary hippocampal neurons *in vitro*. It was observed that both shMaf1-2 and shMaf1-3 lentiviruses could effectively reduce the expression level of Maf1 in hippocampal neurons (Supplementary Fig. 3D). The protein level of Maf1 was also significantly increased following transfection with Maf1-OE lentivirus (Supplementary Fig. 3E). All subsequent viral experiments used shMaf1-3, represented by shMaf1. Notably, it was found that Maf1 was highly expressed in neuronal dendrites and dendritic spines (Supplementary Fig. 3F).

Thus, the primary neurons were extracted and transfected with APP lentivirus, then transfected with shSCR and shMaf1 plasmids to verify this phenomenon *in vitro* in the AD model (Fig. 3F). Consistent with the *in vivo* results, we found that knockdown of Maf1 increased the density of mushroom spines and decreased the density of filopodia/thin-like protrusions *in vitro* (Fig. 3E and F). Overall, the knockdown of endogenous Maf1 expression in hippocampal neurons promoted the growth and development of dendritic spines and increased the density of dendritic spines in neurons under the pathological condition of AD, especially mushroom dendritic spines. Thus, our results suggest that Maf1 plays a vital role in the progression of AD by regulating dendritic spines to regulate synapses, but the specific mechanism still needs further study.

In addition, we injected AAV-syn-shSCR and AAV-syn-shMaf1 into hippocampus of APP/PS1 mice for study. Consistent with phenomenon observed in the Maf1-eCKO1-APP/PS1, knockdown of Maf1 in APP/PS1 mice affected the number and morphology of dendritic spines (Supplementary Fig. 4A–D) and improved learning and memory dysfunction in mice (Supplementary Fig. 4E–G). The effect of Maf1 knockdown in hippocampus was confirmed by immunofluorescence and western blot (Supplementary Fig. 4H and I).

In neural activities, many biological effects, such as the synaptic transmission of nerve cells, transmitter release, cell morphology and growth, and activation of various enzymes are related to calcium signalling pathways. Calcium signalling in dendritic spines plays a significant role in controlling synaptic plasticity.^35^ Previous studies have shown that calcium imbalance may be an important cause of the onset of AD, and the Ca^2+^ level in the endoplasmic reticulum is known to increase in AD and ageing neurons, leading to subsequent compensatory changes and defects in neuronal Ca^2+^ signalling.^36^ Given the accumulating evidence that Ca^2+^ overload can block LTP, leading to synaptic loss and neurodegeneration, we then set out to determine whether Maf1 affects neuronal morphology and function by regulating intracellular Ca^2+^ signalling. The primary hippocampal neurons were extracted and transfected with APP lentivirus, then transfected with shSCR and shMaf1 plasmids. We then used a Fura-4/AM calcium imaging approach to estimate total Ca^2+^ influx (Supplementary Fig. 5A). We found that the Ca^2+^ influx in primary hippocampal neurons was significantly increased under AD conditions. Furthermore, the knockdown of Maf1 decreased the level of Ca^2+^ influx (Supplementary Fig. 5A and B). Thus, our findings suggest that Maf1 can regulate calcium concentration in primary hippocampal neurons.

### MEPSPs and LTP are rescued by Maf1 conditional knockout in hippocampal neurons

Synaptic plasticity is the ability of synapses to respond to increases or decreases in neuronal activity over time. Many studies have suggested that there are potential mechanisms of synaptic plasticity, including changes in the number of neurotransmitters released before synapses and the number of receptors on the postsynaptic membrane, which lead to changes in the efficiency of postsynaptic cells in responding to neurotransmitters. Notably, recording LTP is one of the most commonly used techniques to reflect changes in synaptic plasticity. In addition, mEPSPs reflect spontaneous synaptic activity, referring to the shift in postsynaptic membrane current caused by each vesicle acting on the postsynaptic membrane, indicating the development and maturation of synapses. Previously, we found that Maf1 knockdown can restore the morphology and structure of dendritic spines in APP/PS1 mice. However, can this recovery reverse neurophysiological dysfunction?

**Figure 4:**
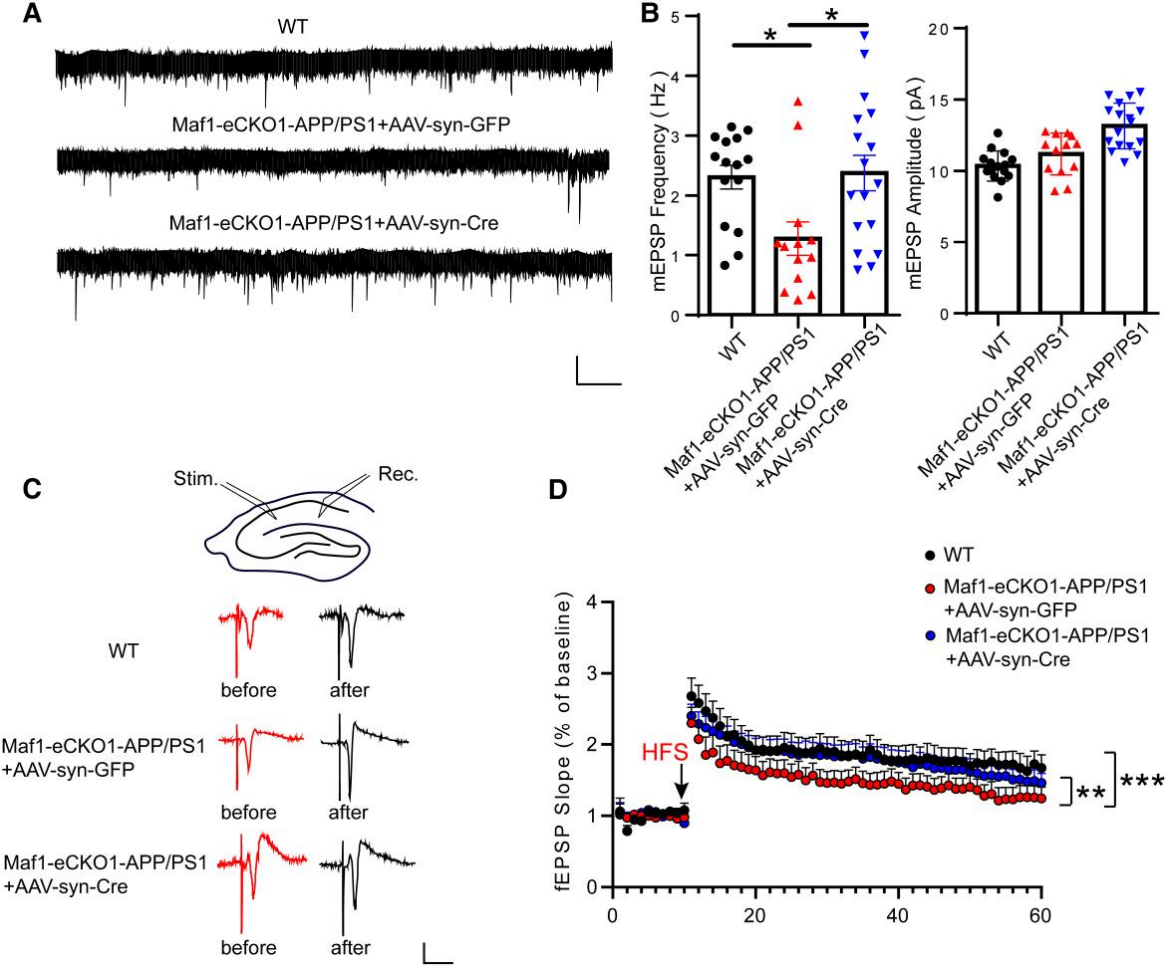
**Conditional knockout of Maf1 rescued synaptic dysfunction in Alzheimer’s disease mice**. (**A**) Micro excitatory postsynaptic potentials (mEPSPs) were recorded in hippocampal CA1 neurons of wild-type (WT) mice and Maf1-eCKO1-APP/PS1 mice injected with AAV-syn-Cre or AAV-syn-GFP. (**B**) The mean mEPSP frequency is shown on the *left* and the mean mEPSC amplitude on the *right*. Calibration: 15 pA, 1 s. Wild-type (WT), *n* = 15 neurons from four mice; AAV-syn-GFP, *n* = 12 neurons from four mice; AAV-syn-Cre, *n* = 12 neurons from four mice. (**C**) Field EPSP (fEPSP) recordings for the CA3-CA1 channel. The stimulation electrode was placed in the Schaffer region of CA3, and the recording pipette was placed in the radiation layer of CA1. The baseline and last 10 min of long term potentiation (LTP) recordings represent fEPSP recordings. Calibration: 0.5 mV, 10 ms. (**D**) Quantitative analysis of the average fEPSP slope in the last 10 min of LTP recordings. Wild-type, *n* = 9 neurons from four mice; AAV-syn-GFP, *n* = 9 neurons from four mice; AAV-syn-Cre, *n* = 11 neurons from four mice. Data are presented as mean ± standard error of the mean. *P < 0.05, **P < 0.01,***P < 0.001.

We further investigated the changes in hippocampal synaptic plasticity in the Maf1-eCKO1-APP/PS1 mice after stereotaxic injection of AAV-syn-GFP or AAV-syn-Cre. To examine synaptic function, we measured mEPSPs in brain sections using whole-cell patch clamp electrophysiology and observed a decrease in their frequency neurons from the Maf1-eCKO1-APP/PS1+AAV-syn-GFP mice, while those hippocampal neurons in the Maf1-eCKO1-APP/PS1+AAV-syn-Cre mice recovered to wild-type levels (Fig. 4A and B). We also examined LTP in the Schaffer collateral-CA1 pathway, a key cellular mechanism for learning and memory. We observed that LTP in the Maf1-eCKO1-APP/PS1+AAV-syn-GFP mice decreased compared with the wild-type mice and recovered in the Maf1-eCKO1-APP/PS1+AAV-syn-Cre mice (Fig. 4C and D). The findings therefore suggested that Maf1 knockdown could restore hippocampal synaptic plasticity.

To further determine whether the increased expression of Maf1 is related to AD pathology, we performed Aβ immunohistochemistry and immunofluorescence staining. We found that, compared with the Maf1-eCKO1-APP/PS1+AAV-syn-GFP mice, Aβ plaques in hippocampus were significantly reduced after Maf1 knockout in the Maf1-eCKO1-APP/PS1+AAV-syn-Cre mice (Supplementary Fig. 4C–F). Then we used ELISA to detect Aβ_40_, Aβ_42_ and β-CTF in hippocampus. We found that the levels of these molecules decreased, accompanied by a decrease in Maf1, after injection of AAV-Cre virus in Maf1-eCKO1-APP/PS1 mice, compared with the Maf1-eCKO1-APP/PS1 mice injected with AAV-GFP (Supplementary Fig. 4G–J). These data suggested that there is a correlation between Maf1 and Aβ.

### Maf1 regulates *Grin1* and may be involved in synaptic function and calcium signalling pathways

**Figure 5:**
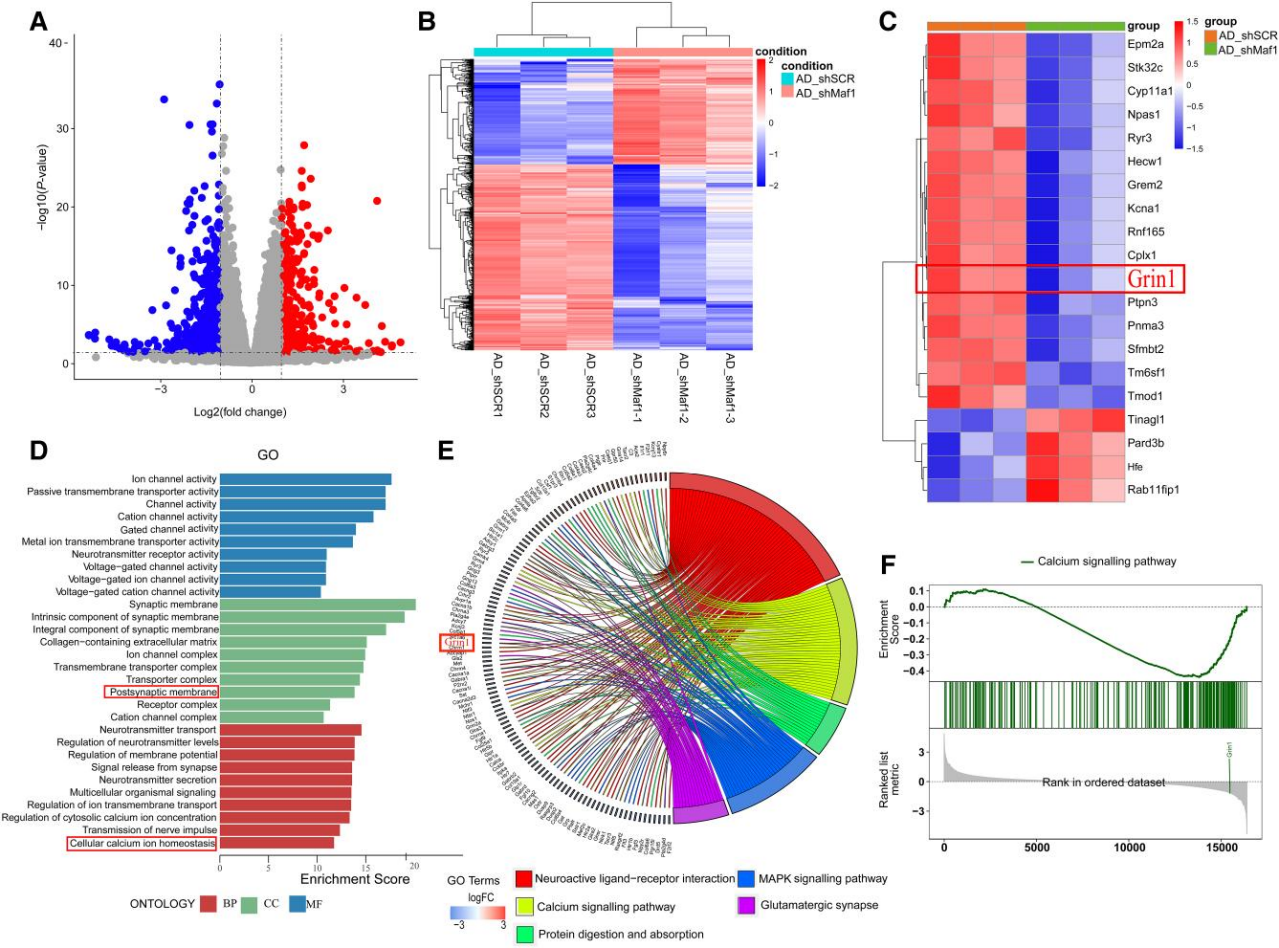
**RNA sequencing analysis suggested that Maf1 regulates downstream gene *Grin1* and may be involved in the calcium signalling pathway and synaptic function**. (**A**) Changes to the neuronal gene expression profile after Maf1 deletion in Alzheimer’s disease (AD) according to RNA sequencing results. The volcano map shows 884 differentially expressed genes, of which 320 were upregulated (red filled circles) and 564 were downregulated (blue filled circles). (**B**) Heat map of differentially expressed genes showing the differences in gene expression profiles between the AD_shSCR (scrambled shRNA sequence) and AD_shMaf1 groups. (**C**) The top 30 entries from a GO enrichment analysis. (**D**) Heat map of differentially expressed genes indicating that *Grin1* was significantly down-regulated after Maf1 deletion. (**E**) KEGG pathway enrichment map of the top five differentially expressed genes. (**F**) Gene Set Enrichment Analysis (GSEA) shows the correlation between the calcium ion signalling pathway and *Grin1*.

We conducted an RNAseq experiment to screen the related genes and signalling pathways that changed after Maf1 was knocked down in AD. Our analysis showed that there were 884 differentially expressed genes after Maf1 was knocked down in AD, of which 564 genes were downregulated and 320 genes were upregulated (Fig. 5A–C). GO enrichment analysis and KEGG pathway analysis of these different genes showed that downregulated genes were mainly related to synapses, calcium ion signalling pathways and nerve-related ligand-receptor responses. In contrast, upregulated genes were primarily associated with immune system diseases, complement and coagulation cascade. We identified genes related to synaptic function and calcium signalling pathway, among which the *Grin1* gene had the best compliance (Fig. 5D–F). Referring to the relevant literature, the *Grin1* gene-encoded protein NMDAR1 is essential in calcium homeostasis and synaptic remodelling in neurons. Therefore, these results suggested that Maf1 may regulate synaptic function by regulating *Grin1* gene expression.

**Figure 6:**
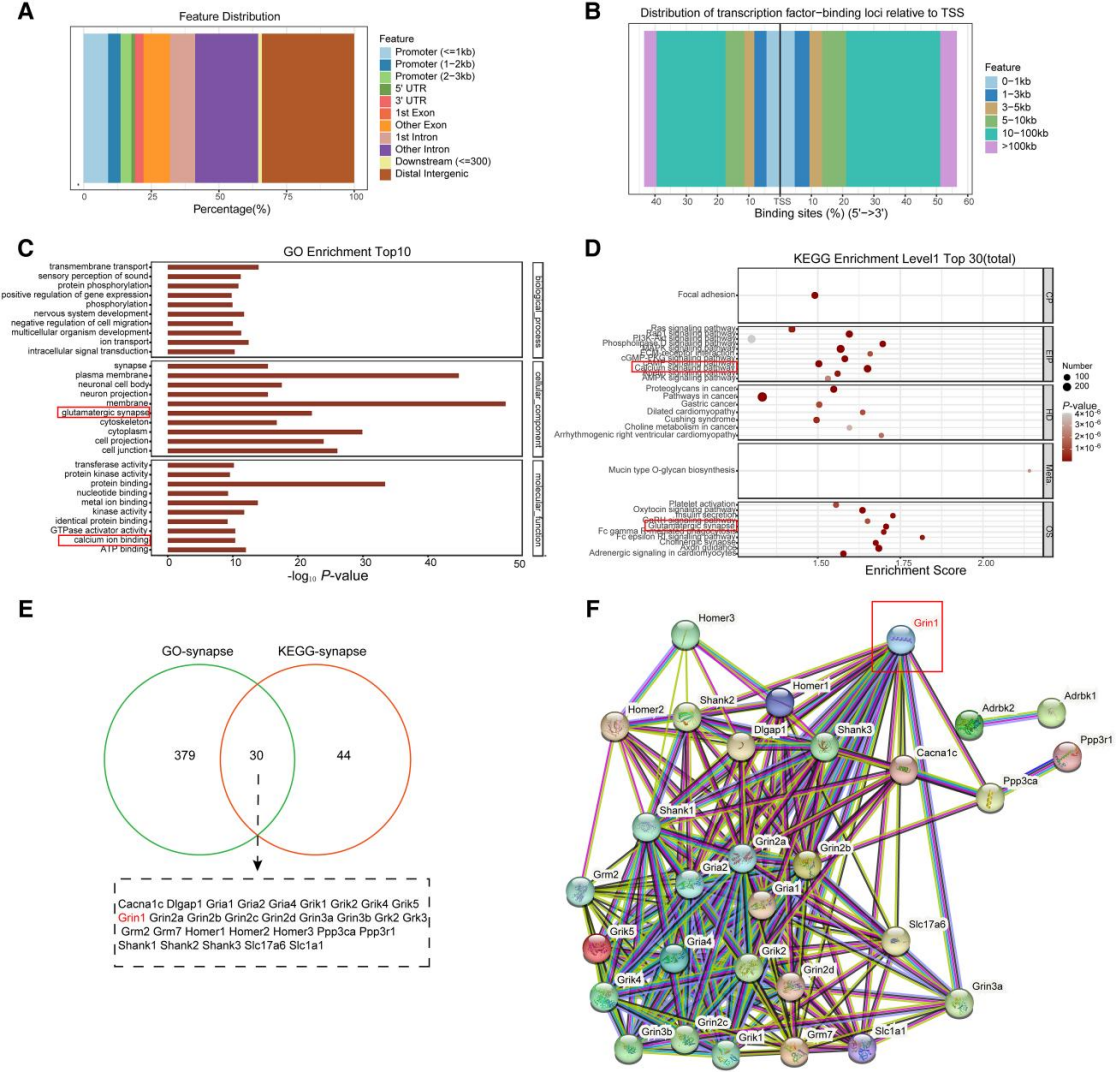
**Cleavage Under Targets and Tagmentation (CUT&Tag) sequencing suggested protein-DNA interaction between Maf1 and *Grin1* gene**. (**A**) Annotated map of Maf1 binding peaks in gene functional elements. (**B**) Map of distance distribution between Maf1 binding peak and gene translation initiation (TSS) site. (**C**) GO enrichment analysis of the top 10 entries of binding site genes, related to synapses and calcium ion binding. (**D**) The top 30 entries in the KEGG enrichment analysis of binding sites showed that calcium signalling pathways and glutaminergic synapses were also closely correlated. (**E**) Schematic diagram of co-intersection genes of GO-synapse and KEGG-synapse enrichment genes. (**F**) Network analysis of protein interaction between GO and KEGG showed that *Grin1* is located at the core.

Cleavage Under Targets and Tagmentation (CUT&Tag) experiments were conducted to further explore the mechanism that underlies Maf1 regulation of synaptic remodelling in AD (Fig. 6A and B). GO analysis suggested a correlation between synapses and calcium ion binding (Fig. 6C), and KEGG analysis suggested a close correlation with calcium ion signalling pathways and glutamatergic synapses (Fig. 6D). GO analysis revealed 409 genes related to synapses and 74 genes related to synapses that were enriched by KEGG pathways. Thirty genes, including *Grin1*, *Grin2a*, *Grin2b* and *Gria1*, co-intersected between them (Fig. 6E), so we further analysed the protein interaction network of these 30 genes. Notably, *Grin1* was located in the core position (Fig. 6F), suggesting that Maf1-NMDAR1 may regulate synaptic function by regulating calcium homeostasis in AD.

### Maf1 regulates NMDAR1 transcription by binding to the *Grin1* promoter region

**Figure 7:**
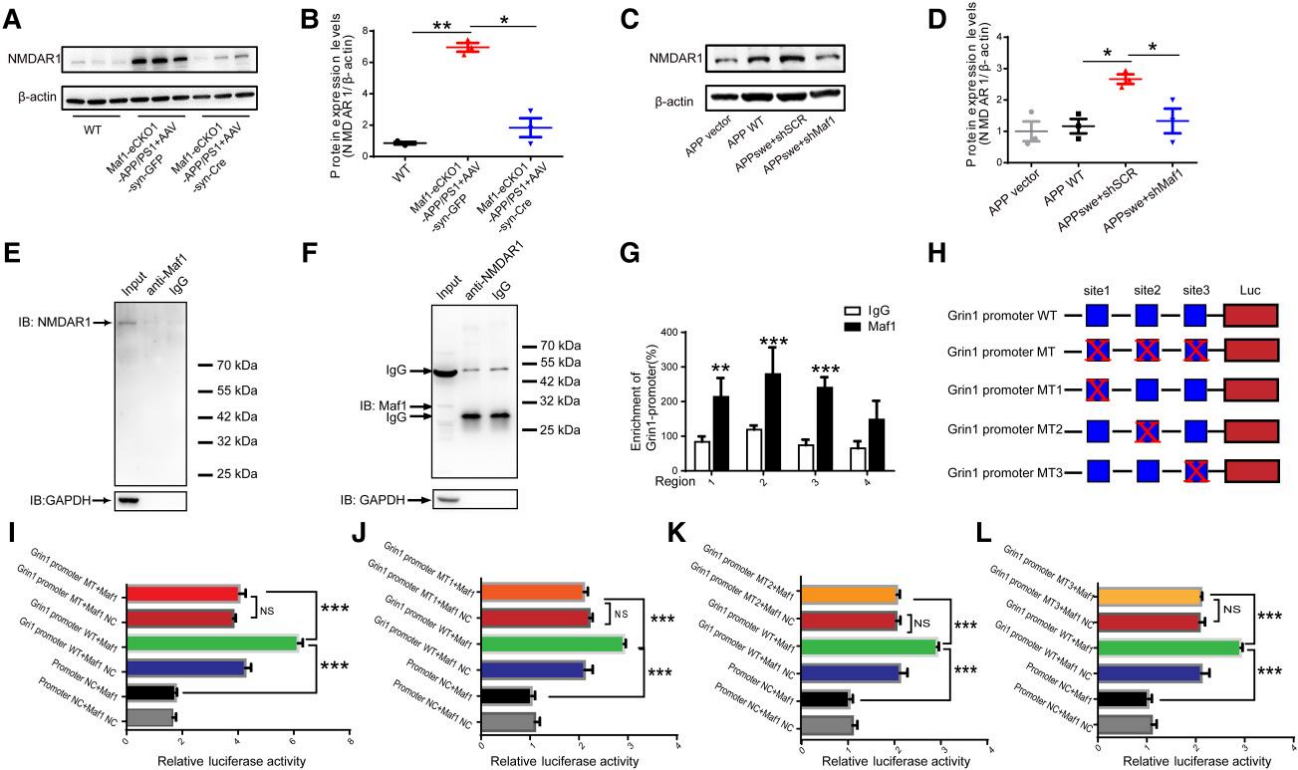
**Maf1 regulates *Grin1* promoter activity and promotes the expression of NMDAR1**. (**A**) Protein levels of NMDAR1 in hippocampus of mice. (**B**) Statistical quantification of western blotting results *in vivo*. (**C**) Protein levels of NMDAR1 in primary neurons. (**D**) Statistical quantification of western blotting results *in vitro*. (**E** and **F**) Immunoprecipitation of Maf1 and NMDAR1 in hippocampal neurons. Maf1 does not bind directly to NMDAR1. (**G**) Four sequences were designed for primer synthesis according to the *Grin1* promoter. The results of chromatin immunoprecipitation (ChIP)-quantitative PCR showed that four sequences in fragments 1, 2 and 3 were enriched in IP-Maf1. (**H**) Schematic construction of wild-type (WT) *Grin1* promoter luciferase (Luc) and mutant (MT) *Grin1* promoter Luc plasmids. (**I**) *Grin1* wild-type and MT promoter plasmids were co-transfected with pcDNA-negative control (NC) and pcDNA-Maf1 into HEK293T cells. Luc activity was detected by using the double Luc reporter gene method. (**J**–**L**) *Grin1* wild-type and mutant promoter plasmids (MT1, MT2, MT3) were co-transfected with pcDNA-NC and pcDNA-Maf1 into HEK293T cells. Data are presented as mean ± standard error of the mean. *P < 0.05, **P < 0.01,***P < 0.001.

Previous sequencing and biogenic analysis suggested that Maf1 might regulate calcium homeostasis and synaptic function by regulating *Grin1* in AD. We found that the level of NMDAR1 protein was downregulated after Maf1 conditional knockout in hippocampus (Fig. 7A and B), which was also verified in primary neurons (Fig. 7C and D). Does Maf1 have regulatory effects on other subunits of NMDAR? We selected NMDAR2A and NMDAR2B for protein expression verification. Protein levels of NMDAR2A and NMDAR2B did not change in hippocampus of the Maf1-eCKO1-APP/PS1 mice injected with AAV-syn-Cre compared with the Maf1-eCKO1-APP/PS1 mice injected with AAV-syn-GFP. This suggested that Maf1 may not have a regulatory effect on the expression of NMDAR2A and NMDAR2B proteins in this study (Supplementary Fig. 6A–C). To clarify the molecular mechanism of how Maf1 regulates the structural remodelling of neuronal dendritic spines through the *Grin1* encoding protein NMDAR1 and thus affects cognitive function, we first verified the protein interaction between Maf1 and NMDAR1 using co-immunoprecipitation (CoIP) assays. We found no CoIP of Maf1 and NMDAR1 proteins from hippocampal neurons (Fig. 7E and F), indicating no protein interaction. Given the gene regulation effect of Maf1 as a transcription factor, we considered that Maf1 might interact with the *Grin1* gene in the nucleus, possibly regulating transcription by regulating *Grin1* promoter activity. To screen the binding sites of Maf1, four primers 2000 bp upstream of the *Grin1* gene were designed with ChIP experiments. The results of ChIP-qPCR confirmed potential binding sites for Maf1 in the *Grin1* promoter region (Fig. 7G).

Subsequently, we performed dual luciferase reporter assays to explore whether Maf1 promotes *Grin1* transcription. We constructed the Maf1 negative control and overexpression plasmids, along with the *Grin1* promoter plasmid and mutated the *Grin1* promoter site to obtain MT, MT1, 2 and 3 (Fig. 7H). Additionally, the luciferase reporter plasmid was used to detect the activity of the *Grin1* promoter in 293T cells. The *Grin1* promoter wild-type and overexpression of Maf1 enhanced the activity of the luciferin reporter gene, suggesting that Maf1 could interact with the *Grin1* promoter and play an enhanced regulatory role at specific sites on the *Grin1* promoter (Fig. 7I–L). Notably, we found that the activity of the mutant *Grin1* promoter luciferase reporter gene was not affected by Maf1 activation (Fig. 7I–L), and there was no difference in the activity of the four mutant plasmid luciferase reporter genes for the *Grin1* promoter, suggesting a synergistic effect between the three sites on the *Grin1* promoter. The results of the ChIP and dual luciferase reporter gene analyses suggested that Maf1 promotes *Grin1* transcription by binding to *Grin1* promoters.

### The morphological effect of Maf1 knockdown is attenuated by *Grin1* promoter mutation

**Figure 8:**
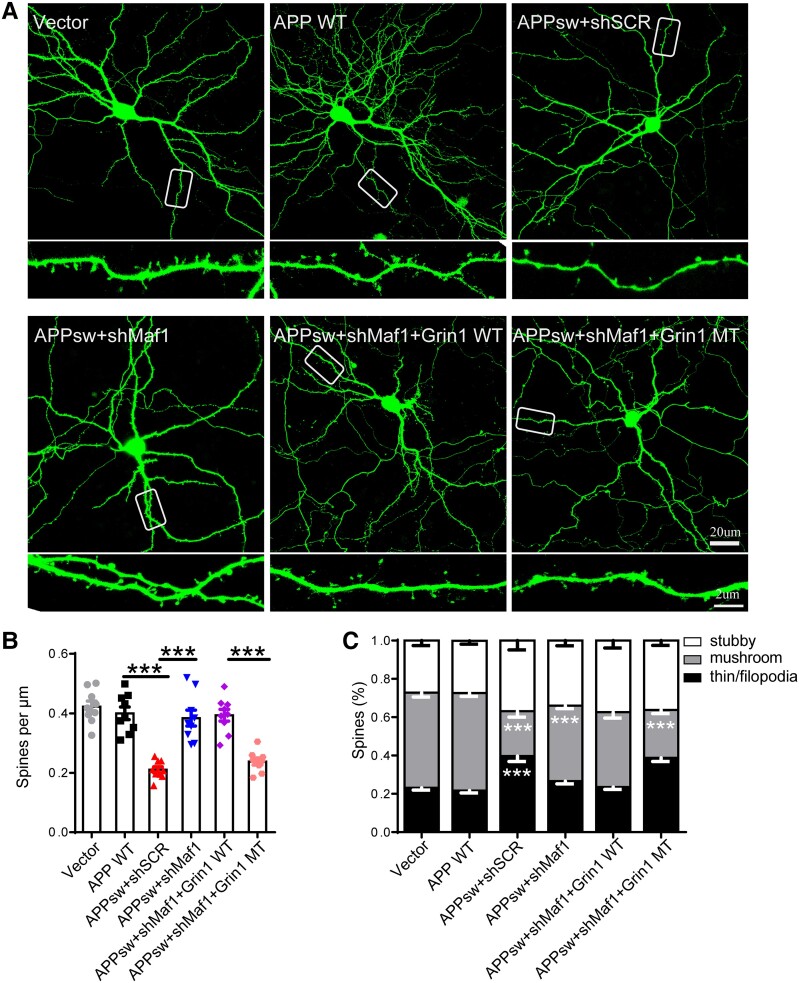
**The morphological effect of Maf1 knockdown was attenuated by *Grin1* promoter mutation**. (**A**) Representative GFP fluorescence images of primary hippocampal neurons after transfection with shSCR, shMaf1, *Grin1* wild-type (WT), *Grin1* mutant (MT) plasmids and APP virus. The areas surrounded by white boxes are enlarged below each image. Scale bars = 20 μm (*top*); 2 μm (*bottom*). (**B** and **C**) Statistical analysis of the density and percentage of classified neuronal dendritic spines *in vitro*. *n* = 7–10 neurons per group. Data are presented as mean ± standard error of the mean. *P < 0.05, **P < 0.01,***P < 0.001.

To confirm the specificity of Maf1 in affecting neuronal dendritic spines through regulation of the *Grin1* promoter, we carried out a ‘rescue’ experiment using a *Grin1* promoter mutation vector. We transfected hippocampal neurons with GFP fluorescent shSCR, shMaf1 and *Grin1* promoter wild-type and mutant plasmids without GFP (Supplementary Fig. 7A), and the morphology and number of neuronal dendritic spines were then analysed. We found that, compared with the shSCR group, the density of neuronal dendritic spines in the shMaf1+ *Grin1* wild-type group was significantly increased, as was the number of mature dendritic spines (Supplementary Fig. 7B and C), which was consistent with the phenotype of Maf1 knockout mice. When the *Grin1* promoter was mutated based on Maf1 knockdown in neurons, it was found that compared with the shMaf1+ *Grin1* wild-type group, the densities of dendritic spines in the *Grin1* mutant groups of neurons were significantly reduced. Furthermore, the number of mushroom dendritic spines decreased (Supplementary Fig. 7B and C), and there was a synergistic effect among the three binding sites, which were involved in the regulation of Maf1 on dendritic spines through the *Grin1* gene. Is this effect still present in the transfected APP virus model? We found that the number of neuronal dendritic spines decreased after transfection with APPswe virus and the proportion of mature dendritic spines decreased, while the morphology and number of neuronal dendritic spines recovered after Maf1 was knocked down, which was consistent with our previous studies (Fig. 8A–C). On this basis, when the region in which Maf1 binds to the *Grin1* promoter was mutated, the regulatory effect of Maf1 on dendritic spines was lost (Fig. 8A–C). In addition, we added NMDAR inhibitors in the case of overexpression of Maf1 and found that Maf1 had no regulatory effect on dendritic spines (Supplementary Fig. 7D–F). Our results confirmed that Maf1 influences the morphology and number of neuronal dendritic spines through its regulatory effect on *Grin1* in AD.

## Discussion

The results of this study have revealed a new mechanism by which the Maf1-NMDAR1 signalling pathway regulates neuronal calcium homeostasis and participates in synaptic remodelling in AD. During AD pathogenesis, the expression of the transcription factor Maf1 increases, which regulates the expression of NMDAR1 by binding to the promoter region of *Grin1*, further regulating calcium homeostasis and changing the morphological structure and number of dendritic spines, thus affecting synaptic function. Ultimately, this regulates synaptic remodelling and affects learning and memory function.

Studies have shown that synaptic structure and function are damaged in the early stage of AD, which is critical to the progression of the disease, and the degree of synaptic damage is positively correlated with the degree of cognitive impairment that ensues.^34^ However, the specific mechanism is still unclear. Recent studies have found that synaptic plasticity is regulated by external signals and internal factors.^3^ Among them, transcriptional regulation, as a decisive factor, can affect the polarization and migration of neurons, the growth and orientation of axons, the growth and branching of dendrites and the generation of synapses.^26^

It has been reported that the transcription factor Maf1 plays a vital role in maintaining the density and structure of synapses under physiological conditions and affects cognitive function,^25^ suggesting that Maf1 may be involved in the pathogenesis of AD. Through the human brain GEO database, we found that hippocampal samples from AD patients showed significantly higher levels of Maf1 than normal elderly controls. It was confirmed that the expression of Maf1 was increased considerably in AD mice and APPswe virus-transfected primary neurons *in vitro*. Previous studies have shown that Maf1 is a transcriptional regulator that can interact with RNA polymerase in the nucleus and that it is involved in the transcription regulation of various RNAs, affecting various metabolic pathways and reducing biosynthetic ability.^21^ We speculate that the increased expression of Maf1 may show toxic effects in AD. A MWM experiment was conducted on wild-type, Maf1-eCKO1-APP/PS1+AAV-syn-Cre and Maf1-eCKO1-APP/PS1+AAV-syn-GFP mice. The results confirmed that Maf1 conditional knockout could improve learning and memory abilities in the AD mouse model, suggesting that Maf1 plays an important role in cognitive function. It was further determined that Maf1 affected the structure and morphology of synapses in AD models *in vivo* and *in vitro*. However, it is still unclear which downstream molecules of Maf1 regulate the morphology and function of neuronal dendritic spines.

A previous study involving gCRND8 mice, a model of aggressive AD amyloidosis, found that NMDAR1 and GluA2 receptor expression was elevated in the early stages of plaque pathology.^37^ Moreover, NMDA receptor subunits and postsynaptic protein PSD-95 in AD were investigated previously, and the study revealed that NMDAR1 and PSD-95 were significantly increased (3–6-fold) in brain specimens of AD patients compared with the control group.^38^ A recent study quantified GluN1 expression in post-mortem hippocampus of AD patients and found increased expression of GluN1 receptor (NMDAR1) in projections of CA1, CA2 and CA3 as well as in the entorhinal cortex.^39,40^ Notably, studies by our group have shown that the expression of NMDAR1 protein in neurons increases after primary hippocampal neurons are transfected with the APPswe virus *in vitro* compared with the control group. The level of NMDAR1 protein expression in APP/PS1 mouse hippocampal neurons was shown to be higher than that of wild-type mice, consistent with the above results from human brain studies. Considering the regulatory effect of Maf1 as a transcription factor on the presence of other genes, we conducted RNAseq and CUT&Tag sequencing to reflect gene expression levels during neuronal maturation. We speculated that Maf1 might regulate calcium homeostasis and synaptic function in AD by regulating *Grin1*. We further demonstrated that Maf1 binds to specific regions of the *Grin1* gene promoter using ChIP and dual luciferase reporter assays, thus playing an enhanced regulatory role in promoter activity.

NMDARs are the main pathway mediating Ca^2+^ signalling in hippocampal neurons and play an important role in excitatory synaptic neurotransmission. Activation of synaptic NMDARs is necessary for synaptic plasticity and the promotion of LTP generation.^41^ However, continued activation of NMDARs can lead to calcium overload and neurotoxicity.^42^ Calcium ions are universal second messengers that regulate many crucial eukaryotic cell functions.^43-45^ Previous studies have shown that calcium imbalance may be a major cause of the onset of AD and that the Ca^2+^ level of the endoplasmic reticulum increases in AD and ageing neurons, leading to compensatory changes and defects in neuronal Ca^2+^ signalling.^36^ Changes in Ca^2+^ signalling alter the balance between Ca^2+^-dependent phosphatase calcineurin (CaN) and Ca^2+^/calmodulin-dependent protein kinase II (CaMKII), which are abundant in synaptic expression. The altered balance of CaMKII and CaN activity blocks LTP, causes synaptic and memory dysfunction and leads to synaptic loss and neurodegeneration.^46-48^ Our hypothesis was based on the ability of Maf1 to regulate *Grin1*, which encodes NMDAR1. We demonstrated that Maf1 can regulate changes in NMDAR1 protein levels. We also determined *in vitro* that Maf1 can affect changes in calcium ion concentration in neuron cytoplasm. Ca^2+^ accumulation in neuronal cells induces the production and deposition of Aβ, leading to impaired learning ability in AD patients.^49^ Simultaneously, we found that Maf1 conditional knockout can affect mEPSP and LTP, suggesting that Maf1 plays an important role in neural activity. Therefore, our results implied that abnormally increased levels of Maf1 promote *Grin1* transcription leading to neurocalcium overload and neuronal overactivity in APP mutant neurons. This suggested that activation of the Maf1-NMDAR1 pathway in AD hippocampus leads to calcium imbalance, which impairs dendritic spine morphogenesis.

The RNAseq results from this study suggested that the immune pathway is upregulated in APP/PS1 animals with downregulated Maf1 expression (Supplementary Fig. 6D–G). From a beneficial perspective, the immune response is ultimately facilitated by the loss of Maf1 expression. Annexin A1 (ANXA1) can reduce Aβ levels by increasing its enzymatic degradation and inhibiting microglia from secreting Aβ-stimulated inflammatory mediators.^50-53^ Studies have shown that C1s and C1r components are key to saving α-secretase activity,^54^ and the complement protein C1q plays an important role in the formation and elimination of synapses in mature neural circuits.^55,56^ In addition, previous investigations have noted that the cognitive and behavioural phenotypes of mice are associated with reduced levels of interleukin-15.^57-59^ CSF1R is a receptor mainly expressed in microglia which regulates microglial activation and survival. Supplementation of CSF1 can improve the deposition of Aβ plaques in 5xFAD mouse brain.^60^ Activated microglia can remove excess apoptotic neurons and repair damage in time to realize the remodelling of brain neurons and astrocyte response in AD. Whether this is beneficial or harmful depends on the types of stimulating factors present when inflammation occurs. Our research focuses on the effects on postsynaptic receptors and calcium ions; although we found that Maf1 may play a role in immune pathways, we will not elaborate more in this paper and more attention will be paid to the role of Maf1 in immune pathways in the future.

In conclusion, our study demonstrates the critical role of the Maf1-NMDAR1 signalling pathway in synaptic and calcium dysfunction in early AD, suggesting that Maf1 may be a potential therapeutic intervention target for early AD. Our study also attempts to reveal a new molecular mechanism related to the pathogenesis of AD. Further research on synaptic dysfunction may open up new ideas and directions for the early intervention and treatment of AD.

## Supplementary Material

awae015_Supplementary_Data

## Data Availability

The datasets used and/or analysed during the current study are available from the corresponding author on reasonable request. Further enquiries can be directed to the corresponding author.
