# Supplementary material for: Maf1 loss regulates spinogenesis and attenuates cognitive impairment in Alzheimer’s disease
Source: Brain. 2024 Jan 16;147(6):2128–43. doi: 10.1093/brain/awae015 (PMC11146433; doi:10.1093/brain/awae015)

Supplementary Figure S1. Maf1 is expressed in hippocampal primary neuronal dendrites.

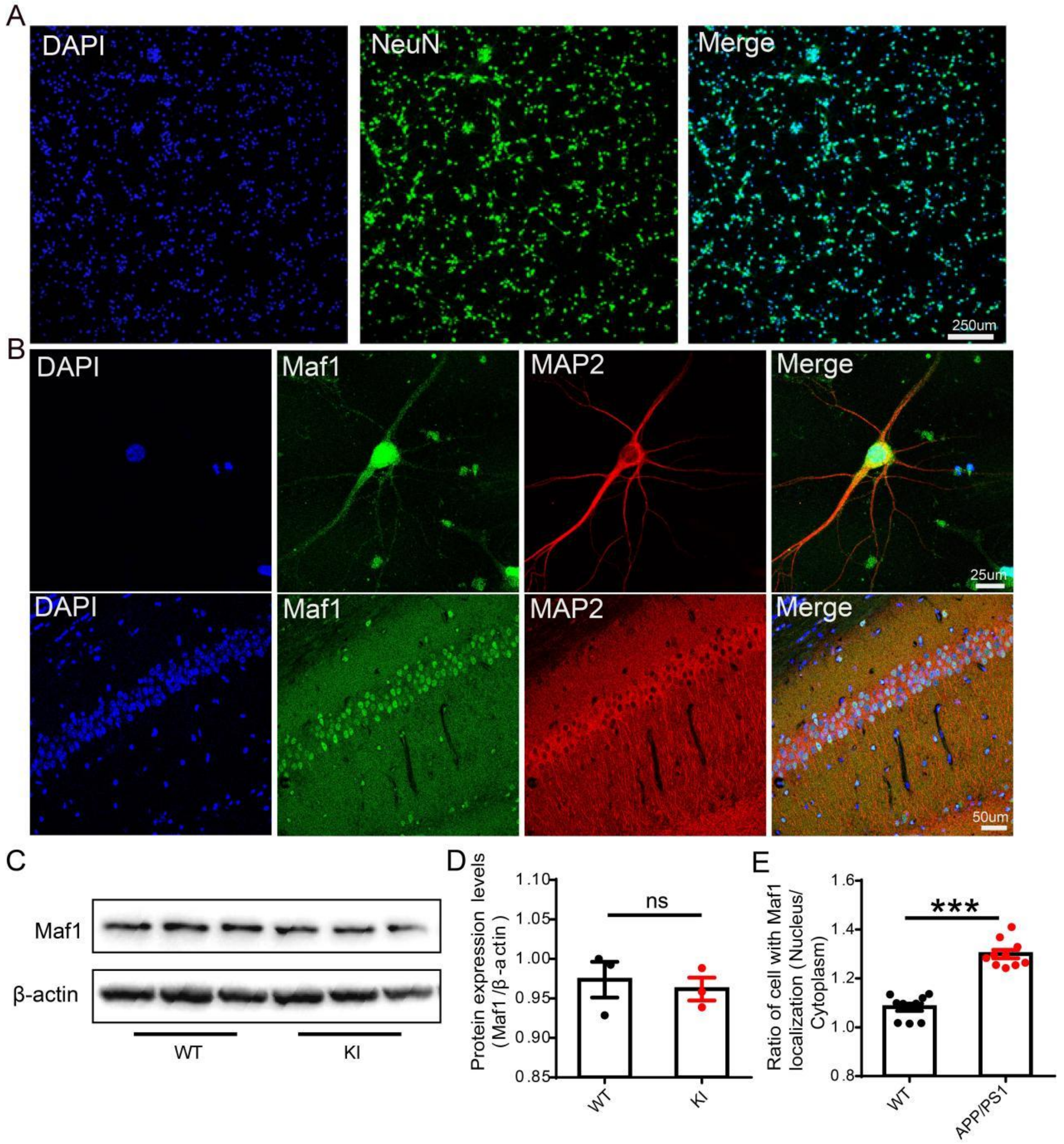

(A) The purity of primary neuron NeuN was observed by laser confocal microscopy. Scale bar: 250 μ m. (B) Laser confocal microscopy was used to observe the immunostaining of Maf1 and MAP2 in 14-day cultured hippocampal neurons and brain slices of 6-month-old mice. Scale bar: above, 25 μm; below, 50 μm. (C) Protein levels of Maf1 in hippocampus of PSEN M146V KI mice. (D) Statistical quantification of Western blotting results. (E) Statistical localization of Maf1 in the nucleus and cytoplasm. Data are presented as the mean ± SEM. $*p < 0.05$; $**p < 0.01$; $***p < 0.001$.

Supplementary Figure S2. Construction of Maf1-eCKO1-APP/PS1 transgenic knockout mice.

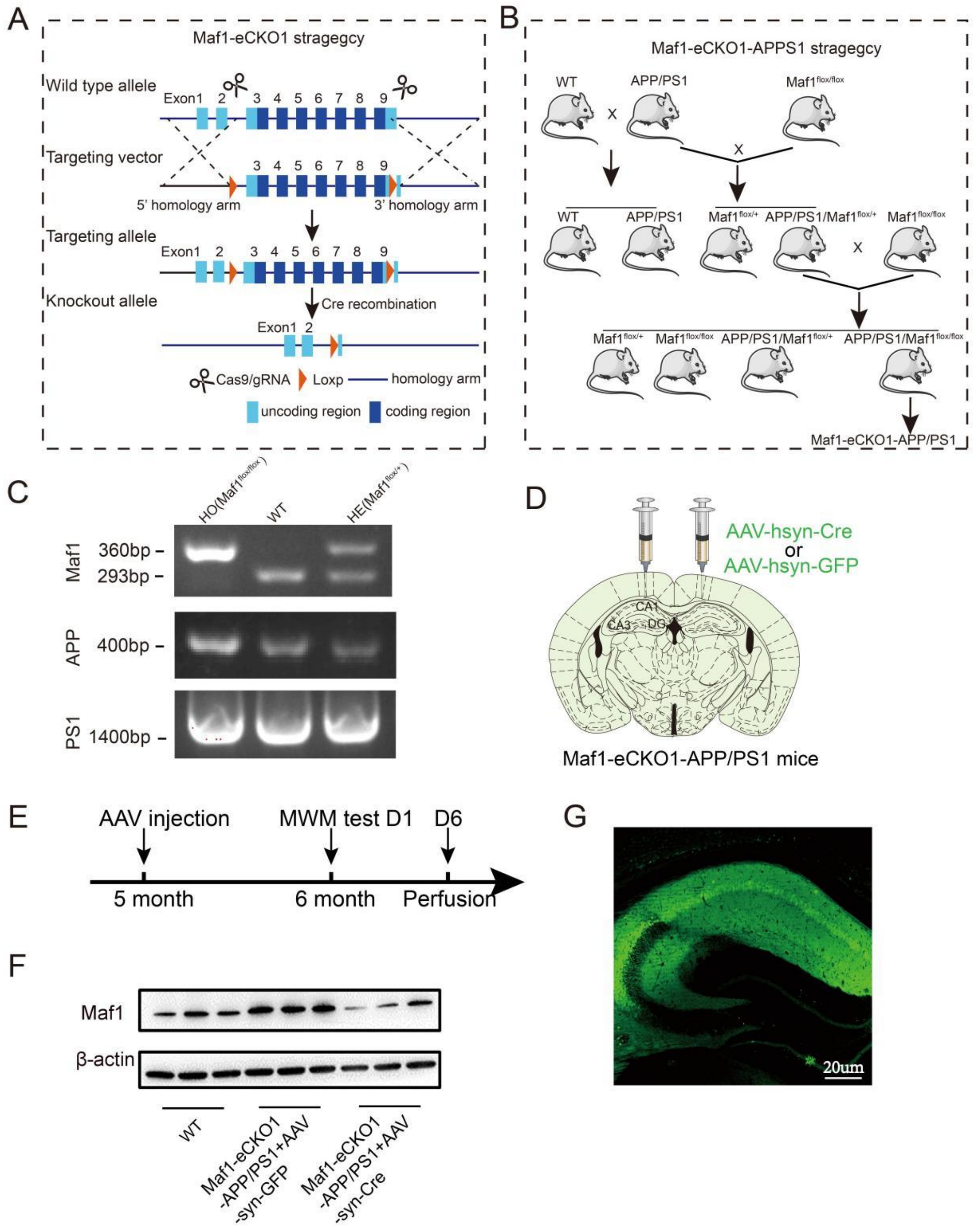

(A) Schematic diagram of construction design strategy for MAF1-eCKO1 conditional knockout mice. Based on the principle of homologous recombination, Maf1 gene was modified by flox through homologous recombination of zygote. (B) Comprehensive scheme of the crossing of APP/PS1 transgenic mice with Maf1 floxed mice. (C) Mice tail PCR identification of Maf1-eCKO1-APP/PS1 hybrid mice. (D) Adeno-associated virus stereotactic injection pattern. (E) Schematic of experimental design. Viral

injections were performed on 5-month-old mice and Morris water maze (MWM) test (same as Supplementary Figure S4) were performed after 1 month. (F) Western blotting was used to confirmed the successful knockdown of Maf1 and the corresponding decrease of NMDAR1 in Maf1-eCKO1-APP/PS1 transgenic mice after injecting AAV-Cre virus. (G) Fluorescence map of AAV-Cre virus injected into hippocampus of Maf1-eCKO1-APP/PS1 hybrid mice. Scale bar: 20 μm.

Supplementary Figure S3. Construction of interfering shRNA virus and full-length virus of target gene Maf1 and verification in cell lines and primary neurons.

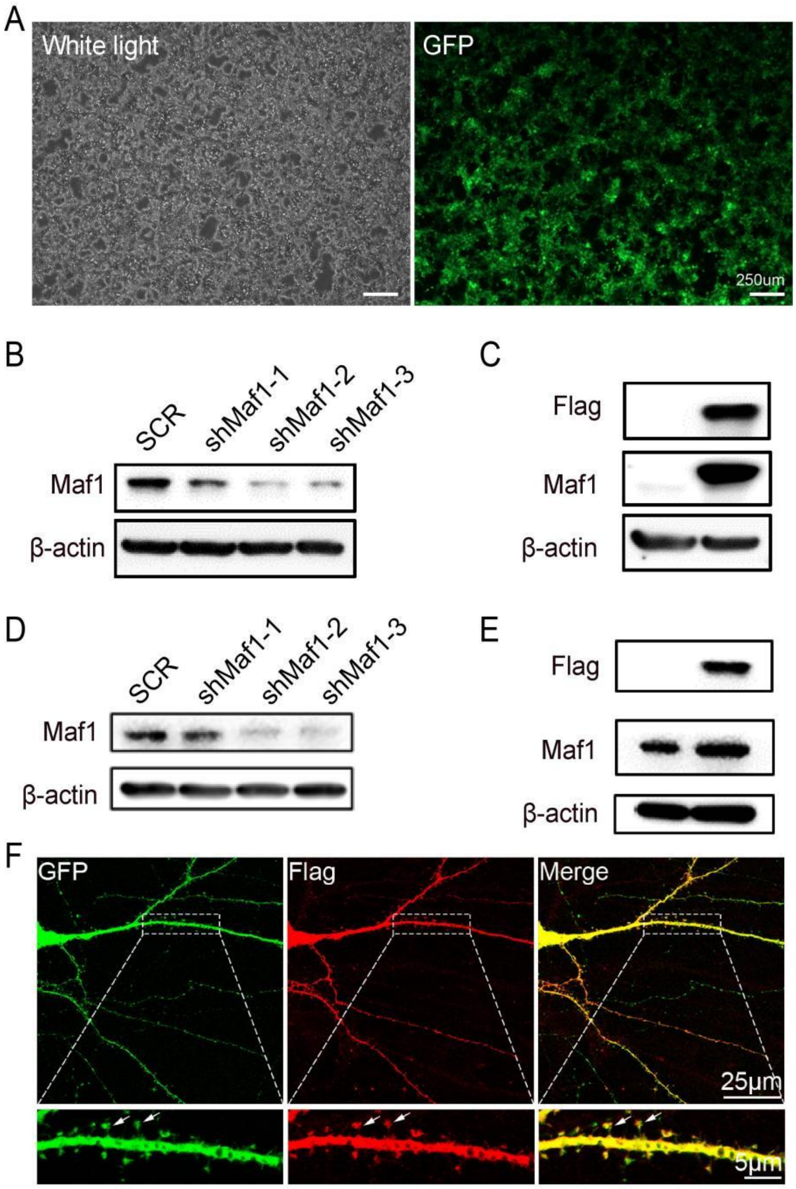

(A) The virus was transfected into 293T cell lines, and the green fluorescence intensity of GFP was higher 72 hours after transfection. (B-C) The knockdown effect of shRNA on Maf1 and the effect of Maf1 overexpression in 293T cell lines were detected by western blotting. (D-E) The knockdown effect of shRNA on Maf1 and the effect of Maf1 overexpression in primary neurons were detected by western blotting. (F) Maf1 overexpression plasmid with Flag label was transfected into neurons, and immunofluorescence staining was performed after 14 days of culture.

Supplementary Figure S4. Knockdown of endogenous Maf1 in hippocampal neurons improved cognitive function in AD.

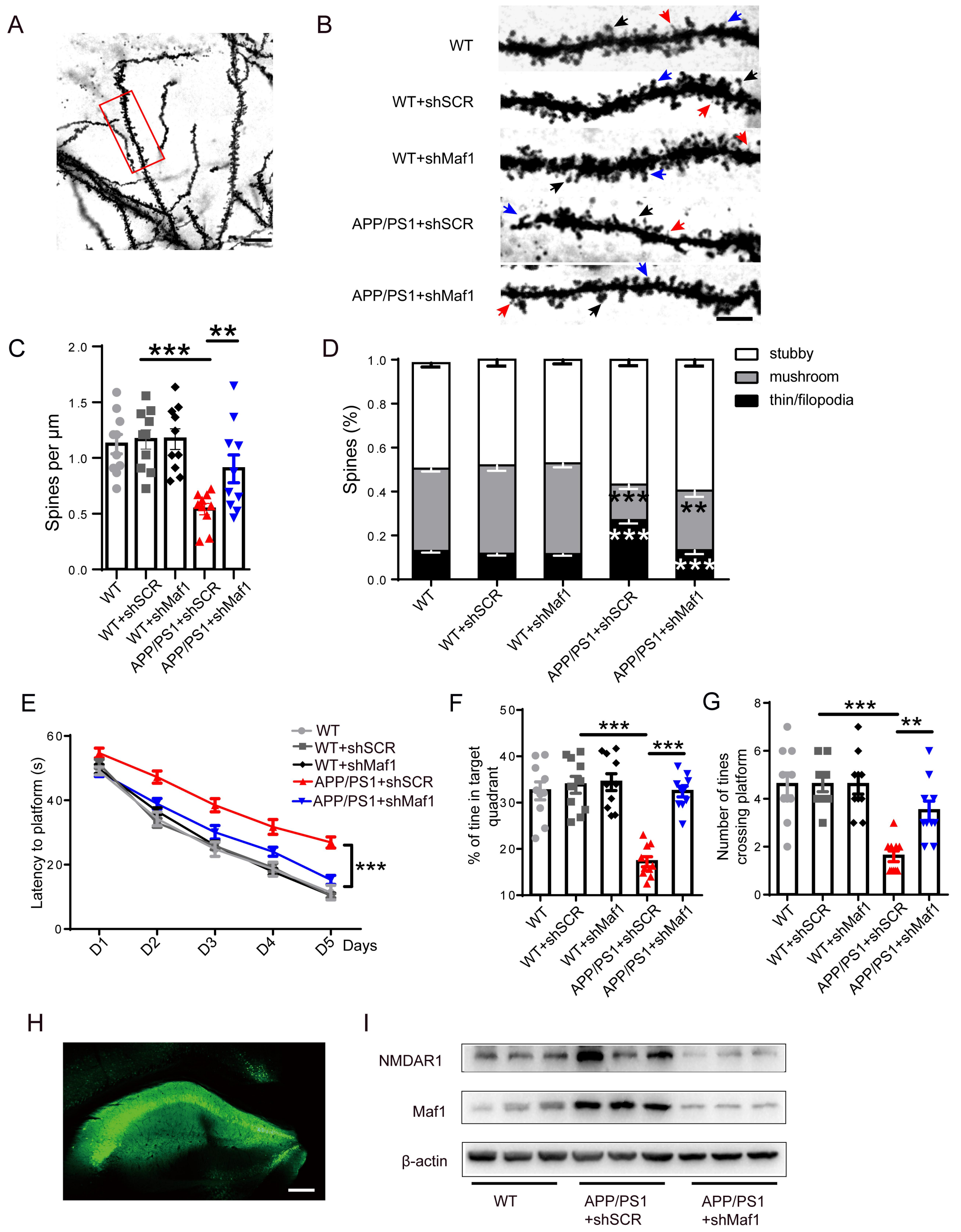

(A) Golgi staining of mice hippocampal neurons. The neurons were selected under 40x microscope, and the morphology and quantity of dendritic spines were analyzed under 100x microscope. Scale bar, 25μm. (B) Representation of hippocampal neuronal dendritic spines in WT mice and APP/PS1 mice injected with

AAV-syn-shSCR and AAV-syn-shMaf1 at high power after Golgi staining. Scale bar, 10 μm. (C) Statistical analysis of the density of neuronal dendritic spines in the above groups of mice. (D) Statistical diagram of classified percentage of neuronal dendritic spines in the above groups of mice. n=10 neurons for each group. (E) The latency to find the escape platform were measured on days 1–5 of the acquired training phase. (F-J) The percentage of time in the target quadrant and the number of times of crossing the target platform area in the space exploration phase of the above groups of mice. n=10 mice for per group. (H) The expression of AAV-GFP green fluorescence was observed in the hippocampus of mice in brain slices 1 month after virus injection. (I) Western blotting was used to detect the expression of Maf1 in the hippocampus of AD mice injected with AAV-shMaf1. Data are presented as the mean ± SEM. *$p < 0.05$; **$p < 0.01$; ***$p < 0.001$.

Supplementary Figure S5. Knockout of Maf1 can affect calcium homeostasis and amyloid plaque deposition in hippocampal neurons of AD.

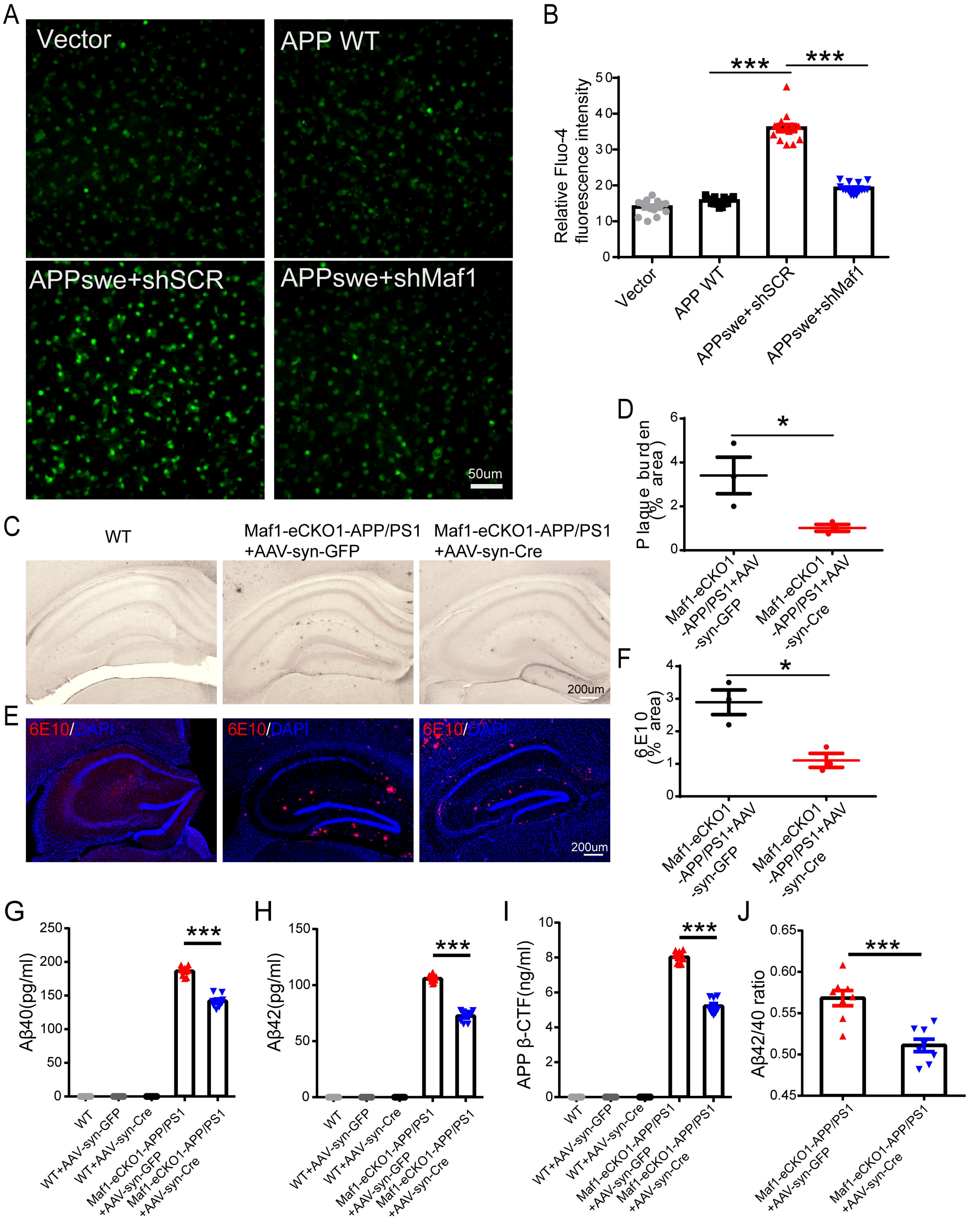

(A) Primary hippocampal neurons were cultured in vitro after infection with APP, shSCR and shMaf1 lentivirus. The green fluorescence under fluorescence microscope represents Fura-4/AM intensity. (B) Statistical quantification plots of the resulting fluorescence intensities for the four groups. (C-D) Immunohistochemistry and statistical result of Aβ plaques in Maf1-eCKO1-APP/PS1 mice at 6 months. (E-F)

Immunofluorescence and statistical result of Aβ plaques in Maf1-eCKO1-APP/PS1 at 6 months. (G-I) The levels of Aβ40, Aβ42 and β-CTF in mice hippocampal by ELISA kits. (J) The ratio of Aβ42 and Aβ40 in mice hippocampal. *$p < 0.05$; **$p < 0.01$; ***$p < 0.001$.

Supplementary Figure S6. Effects of Maf1 knockdown on NMDAR subunits and regulation of immune pathways.

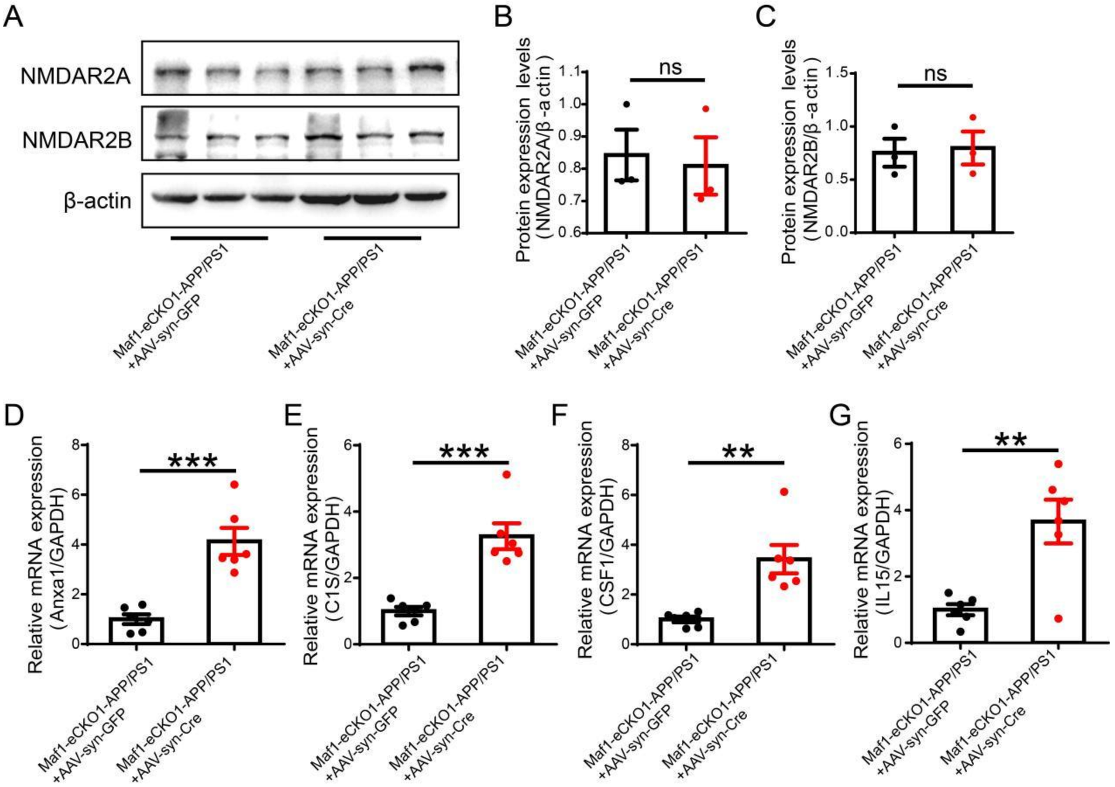

(A) Protein levels of NMDAR2A and NMDAR2B in hippocampus of mice; (B-C) Statistical quantification of Western blotting results *in vivo*. (D-G) Statistical changes of mRNA levels of immune pathway factor Anxa1, C1S, CSF1 and IL15 after Maf1 knockout in hippocampus. Data are presented as the mean ± SEM. $*p < 0.05$; $**p < 0.01$; $***p < 0.001$.

Supplementary Figure S7. The morphological effect of Maf1 knockdown was attenuated by Grin1 promoter mutation and NMDAR inhibitor.

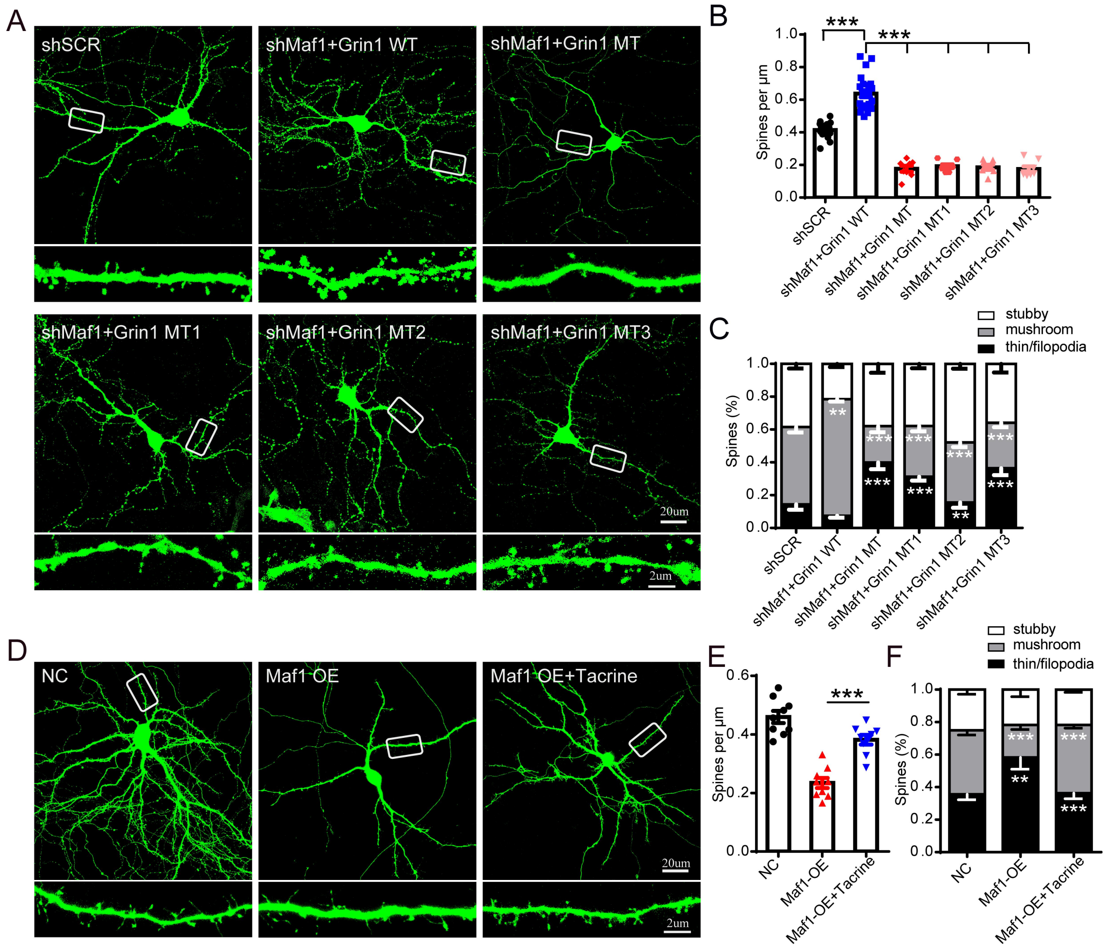

(A) Representative GFP fluorescence images under fluorescence microscope of primary hippocampal neurons after transfection with shSCR, shMaf1 and Grin1WT and MT plasmids. Below is local magnification in white box. Scale bar: above, 20 μm; below, 2 μm. (B-C) Statistical analysis of the density and percentage of classified neuronal dendritic spines in vitro. (D) Representative GFP fluorescence images of primary hippocampal neurons after transfection with NC and Maf1 OE plasmids, and Tacrine hydrochloride traetment (NMDAR inhibitor). Below is local magnification in white box. Scale bar: above, 20 μm; below, 2 μm. (E-F) Statistical analysis of the density and percentage of classified neuronal dendritic spines in vitro. *n*=7~20 neurons for each group. Data are presented as the mean ± SEM. *$p < 0.05$; **$p < 0.01$; ***$p < 0.001$.

Supplementary Table S1

Primer sequences used for PCR in this study.

Primer		Sequences
Anxa1	Forward	ATGTATCCTCGGATGTTGCTGC
	Reverse	TGAGCATTGGTCCTCTTGGTA
C1S	Forward	GCTGCTCACGTTTTGGAGAAA
	Reverse	ACGTTTACTGTAGAGTCTCTGGG
CSF1	Forward	ATGAGCAGGAGTATTGCCAAGG
	Reverse	TCCATTCCCAATCATGTGGCTA
IL15	Forward	ACATCCATCTCGTGCTACTTGT
	Reverse	GCCTCTGTTTTAGGGAGACCT
Grin1-promoter-F1	Forward	GCCCAAGAAGAGCCCAAGAA
	Reverse	CCAGGGAAGATTTGTCACGC
Grin1-promoter-F2	Forward	GACAGGGGTCTTAGACAGTGC
	Reverse	ACCCACACGTTCACACTCC
Grin1-promoter-F3	Forward	CTTTACATGGCAGGAGGGGG
	Reverse	CATCTTGCTCCCAGCAGTCA
Grin1-promoter-F4	Forward	CGGATCCAAGGTGTGGTGTT
	Reverse	TCTAGAGGGTGAGATAGGATTGCT

Supplementary: Unmodified full-length gels/blots

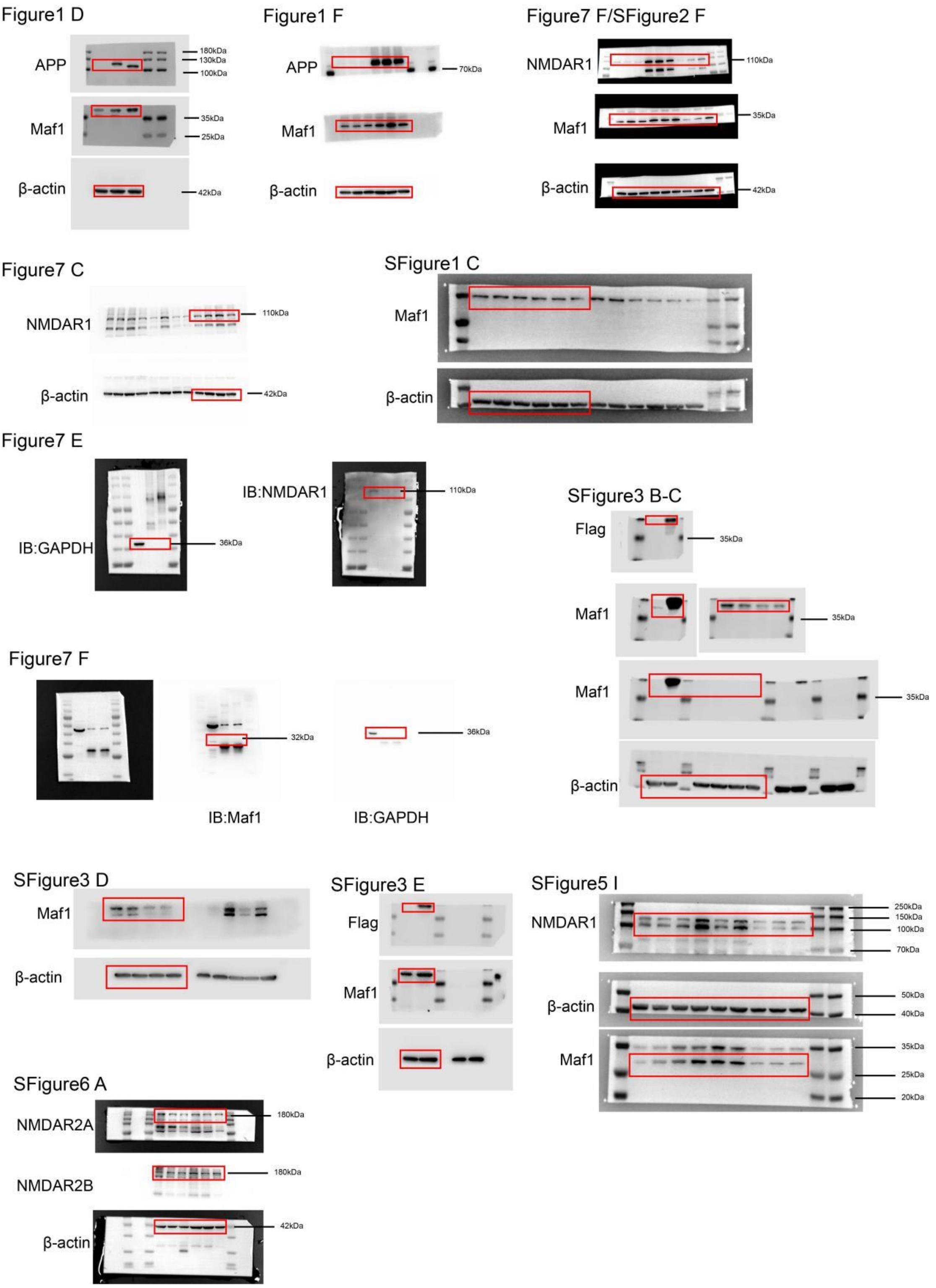

Supplement: awae015_Supplementary_Data [file awae015_supplementary_data.pdf]
